# Splice Isoforms of the Apoptosis Gene *BCL-X* Have Opposing Effects in Diabetic Kidney Disease: Potential Treatment Target and Prognostic Value

**DOI:** 10.1155/jdr/2840106

**Published:** 2026-03-26

**Authors:** Megan Stevens, Monica L. Ayine, Kim Gooding, Angela Shore, Pedro Marqueti, Sebastian Oltean

**Affiliations:** ^1^ Department of Clinical and Biomedical Sciences, University of Exeter Medical School, Exeter, UK, exeter.ac.uk; ^2^ NIHR Exeter Clinical Research Facility, Royal Devon University Healthcare Foundation Trust, Exeter, UK

**Keywords:** alternative splicing, apoptosis, *BCL-X*, diabetic nephropathy, therapeutic targets

## Abstract

In recent years, the importance of alternative splicing (AS) of certain genes in the nature and progression of diabetic nephropathy (DN) has been studied. We report a novel AS event observed in the diabetic kidney—AS of the apoptosis gene *BCL-X* to increase the proapoptotic *BCL-XS* and decrease the antiapoptotic *BCL-XL*. This study is aimed at further investigating the role of this novel AS event in the pathogenesis of DN. To characterize important splicing events in progression of DN, human glomerular endothelial cells (GEnCs) were exposed to a diabetic environment for 1 week, and RNAseq was performed. Although several splicing events were discovered, we focused on the apoptosis gene *BCL-X* in the present study. In GEnCs, an upregulation of the *BCL-XS/BCL-XL* ratio was observed, resulting in an increase in GEnC apoptosis. An upregulation of *IL-6* was also observed, and treatment with IL‐6 alone induced a dose‐dependent shift in *BCL-X* splicing to promote expression of the proapoptotic *BCL-XS*. Furthermore, we identified certain splicing factors, SF3B1 and PTBP1, involved in *BCL-X* splicing regulation. Overexpression of the antiapoptotic *BCL-XL* isoform rescued apoptosis, suggesting a possible therapeutic avenue. In patients, an increase in the proapoptotic *BCL-XS* in urinary RNA correlated with a decline in the glomerular filtration rate, whereas in blood it correlated with the level of albuminuria. There is an increase in the proapoptotic *BCL-XS* isoform in the diabetic glomerular endothelium, resulting in increased GEnC apoptosis. Increased *BCL-XS* expression correlates with markers of renal function decline, implicating this AS event as a potential biomarker for DN severity. Furthermore, a switch of isoforms from *BCL-XS* to *BCL-XL* is highlighted as a novel therapeutic strategy.

## 1. Introduction

With the increasing incidence of diabetes worldwide, there has been an increase in diabetes‐associated complications, including diabetic nephropathy (DN). A large proportion of patients with DN will eventually progress to end‐stage renal failure and require long‐term kidney replacement treatment (dialysis). This has placed a considerable and ever‐increasing strain on health system resources worldwide. Therefore, there is a clear clinical need for new therapeutic strategies that can slow the progression of DN [1–3].

At present, few therapeutic tools are available to clinicians for the treatment of DN. Blood pressure lowering angiotensin‐converting enzyme (ACE) inhibitors have long been proven to be beneficial in decreasing proteinuria and slowing the degradation of kidney function [4, 5]. Furthermore, more recently, SGLT2 inhibitors, designed to lower glycemia, have shown remarkable effects on the protection of kidney function [6, 7]; although the mechanism is not yet completely understood, it is clearly not due only to decreasing glycemia. However, the above‐mentioned drugs, although extremely useful, were not purposely designed for the treatment of DN; their effects were discovered serendipitously. Therefore, understanding the molecular mechanisms involved in the progression of DN and designing drugs to directly address these mechanisms has the potential to elicit new classes of drugs with good efficiency in slowing the progression of DN [8].

One new area of therapeutic research in various diseases is the modulation of alternative splicing (AS). AS occurs in over 94% of human genes and therefore represents an important step in deciding the proteome diversity of the functional capabilities of cells [9, 10]. Since AS is so widespread, it is not surprising that virtually all diseases display aberrant splicing, which includes the appearance of new, specific disease isoforms or modifications to the ratios of existing isoforms [11, 12]. Either way, AS has the potential to maintain and drive the pathogenic process. Therefore, a new research field is looking into the manipulation of these isoforms for therapeutic benefit, for example, by switching ratios of splice isoforms to their normal rapport [13].

To explore these therapeutic AS decisions further, we need to understand the potentially pathogenic isoforms. Therefore, this study is aimed at determining important AS changes that occur in the progression of DN. We describe in detail one such splicing event—the pro‐ and antiapoptotic splice isoforms of *BCL-X* (an apoptosis gene). We describe its regulation in a diabetic environment, the association of isoform ratio with DN progression, and its potential as a therapeutic target in DN.

## 2. Materials and Methods

### 2.1. Cell Culture and Treatments

Previously characterized conditionally immortalized glomerular endothelial cells (GEnCs) [14] were differentiated in endothelial media (EGM + Bulletkit; Lonza) for 5 days. Prolonged culture of kidney cells in vitro can promote insulin receptor degradation; therefore, to mimic a diabetic environment and induce cellular resistance in vitro, treatments were performed with a “glucose soup” (GS: 25 mM glucose, 1 ng/mL TNF‐*α*, 1 ng/mL IL‐6, and 100 nM insulin), as previously reported [15–17]. Controls included normal glucose (NG: 5.5 mM glucose) or an osmotic control, mannitol (MAN: 5.5 mM glucose and 19.5 mM mannitol). Treatments were performed in growth factor deficient EGM media with reduced fetal bovine serum (FBS: 0.5%) (“TREAT” media) daily for 7 days. GEnCs were also treated with varying concentrations of IL‐6 (0–50 ng/mL) for 6–24 h in TREAT media.

HEK293 cells (ATCC) were subcultured from existing cultures in the lab in low glucose DMEM (Sigma‐Aldrich) supplemented with 10% FBS or reduced to 0.5% for treatment and 1% pen/strep antibiotics (Sigma‐Aldrich). HEK293 cells were treated in either normal glucose (NG) containing 5.5 mM glucose, high glucose (HG) containing 25 mM glucose, GS+ (containing IL‐6), (MAN), or GS‐ (no IL‐6). The oscillating treatment involved changing media from NG to either M, HG, GS+, or GS− back and forth every 12 h till 48 h is reached. Cell lines were genotyped for authentication. Mycoplasma detection was routinely assessed in our labs.

### 2.2. Rnaseq With Gene Expression and AS Analysis

RNA extraction from GEnCs treated for 1 week with either GS or MAN was performed using the RNAeasy kit (Qiagen). RNA concentration and quality were determined using the TapeStation (Agilent).

High depth and coverage Next Generation Sequencing (NGS) analysis was performed using the Illumina HiSeq 2500 platform (Exeter Sequencing Service). We used 125 bp paired end reads at a high depth and coverage of 125 million reads per sample. Each condition, GS and MAN, had five replicates. Reads were quality‐filtered and adapter trimmed using FastQC from the Galaxy bioinformatics platform package. Hisat2, which is able to detect splice junctions, was used to align reads against the reference genome, Hg38. Htseq was used to calculate gene counts before differential analysis using DESeq2. Genes with an FDR (*q* value) < 0.05 were deemed significantly altered.

MISO (mixture‐of‐isoforms) is a probabilistic framework that was used to quantify the expression level of alternatively spliced genes from our RNAseq data, which was performed by AccuraScience (https://www.accurascience.com/). We implemented a *Δ*PSI (Percentage Splicing Index) cutoff of 0.2 and Bayes factor cutoff of 10 to select significantly (AS) events between the GS and MAN treated GEnCs.

### 2.3. RT‐PCR

RNA was extracted from treated GEnCs using the RNAeasy kit (Qiagen), per the manufacturer instructions. cDNA was synthesized from the RNA using the GoScript Reverse Transcription System (Promega). RT‐PCR for *BCL-X* splice isoforms was performed using a forward primer positioned in exon 2, before the 5^′^ splice site for the *BCL-XS* isoform, and a reverse primer positioned in exon 3: *BCL-X* F, 5^′^‐CATGGCAGCAGTAAAGCAAG‐3^′^ and R, 5^′^‐GCATTGTTCCCATAGAGTTCC‐3^′^. The PCR program consisted of 95°C for 120 s, 35 cycles of 95°C for 30 s, 55°C for 30 s and 72°C for 60 s, followed by 72°C for 10 min. A band sized ~175 bp denoted *BCL-XS,* whereas a band sized ~355 bp denoted *BCL-XL*. A reverse transcriptase negative (RT‐) and water control was used for all PCR reactions. PCR products were run on the Biolanalyzer (Agilent) to enable quantification of *BCL-XS* and *BCL-XL* products. The primer sequences for other targets (*ZNF426, ARAP1,* and DOK1) are shown in Table S1.

Quantitative RT‐PCR (qRT‐PCR) was used to assess changes in gene expression using 20 ng cDNA per reaction. The primers are detailed in Table S1. SYBR green dye was used with the following PCR conditions using the LightCycler (Roche): 95°C for 30 s, followed by 40 cycles of 95°C for 1 s and 59°C for 20 s. Relative quantification analysis was performed by normalizing the gene of interest to a housekeeping control (*HPRT1*) following the *ΔΔ*Cq method.

### 2.4. Western Blotting

Denatured protein samples were run on mini‐PROTEAN TGX Stain Free precast gels (4%–15%, BIORAD), which allow for visualization and accurate analysis of the total protein loaded for each sample using a Gel‐Doc EZ (BIO‐RAD) imaging system. The use of this system means a housekeeping protein loading control is not required as the amount of protein on the membrane for each sample can be quantified. Once the proteins had been transferred on to a polyvinylidene difluoride (PVDF) membrane, total protein could be quantified. Membranes were blocked in 3% bovine serum albumin (BSA) in tris‐buffered saline (TBS) plus 0.3% Tween before being probed with anti‐BCL‐XS/L (Santa Cruz) at 1:1000 dilution in 3% BSA‐TBS‐Tween (0.3%), at 4°C overnight. After washing membranes in TBS‐Tween (0.3%), they were incubated in goat antimouse fluorescent secondary antibody (LI‐COR) diluted in 3% BSA‐TBS‐Tween (0.3%), 1:10,000. Membranes were washed again and imaged with the LI‐COR Odyssey CLx. Analysis was performed using the Image Studio software (LI‐COR).

### 2.5. Immunofluorescence

GEnCs plated onto coverslips were fixed with 100% methanol for 5 min before blocking with 3% BSA and 5% normal goat serum in phosphate‐buffered saline (PBS) for 1 h. Cells were then incubated with anti‐SF3B1 (Abcam) at 1:100 dilution in 3% BSA in PBS for 1 h at room temperature. After washing in PBS, the appropriate fluorescent secondary antibody was used (Alexa Fluor) in 3% BSA in PBS for 2 h at room temperature. Sections were then washed in PBS before mounting with gel mount containing DAPI (VECTASHIELD). Images were taken using a Leica DM4000 B LED fluorescent microscope using a 40× objective.

### 2.6. Apoptosis Assays

*JC-10 mitochondrial membrane potential assay (Abcam):* This assay detects changes in the mitochondrial membrane potential using the cationic, lipophilic JC‐10 dye. In normal cells, JC‐10 concentrates in the mitochondrial matrix where it forms red fluorescent aggregates. However, in apoptotic and necrotic cells, JC‐10 diffuses out of the mitochondria, changes to monomeric form, and stains cells with green fluorescence. We display the data as the mitochondrial polarization relative to control as a marker of apoptosis. Assays were performed on GEnCs and HEK293 cells plated and treated in a 96‐well format in triplicate, per the manufacturers′ protocol.

*Generic caspase activity assay (abcam):* This assay measures the activity of caspase, a widely accepted reliable indicator for cell apoptosis, using TF2‐VAD‐FMK as a fluorescent indicator that irreversibly binds to activated caspase‐1, ‐3, ‐4, ‐5, ‐6, ‐7, ‐8, and ‐9 in apoptotic cells. Assays were performed on GEnCs plated and treated in a 96‐well format in triplicate, per the manufacturer′s protocol.

*Trypan blue cell viability assay (ThermoFisher Scientific):* Trypan blue stain colors dead cells blue, allowing cell viability to be measured. Assays were performed in GEnCs plated and treated in a 12‐well format, per the manufacturer′s protocol.

### 2.7. Transfections

The transfection was performed on HEK293 cells seeded in a black, clear bottom 96 well culture plate, at a density of 2 × 10^4^ cells per well in a complete growth medium, overnight. The transfection complex comprised 0.2 *μ*g/*μ*L of the plasmid DNA diluted in serum‐free medium (Opti‐MEM) and gently mixed with Turbofectin 8.0 (Fisher Scientific) at a factor of 3:1. The mixture was incubated for 15 min at room temperature, gently added to the cells, and evenly distributed by carefully rocking.

### 2.8. Human Samples and Ethics Approval

The samples were obtained from participants with and without Type 2 diabetes taking part in the Biomarker Enterprise to Attack Diabetic Kidney Disease (BEAt‐DKD) Exeter and VIBE study in the NIHR Exeter Clinical Research Facility. The samples were only used for purposes identified by the research protocols for which appropriate ethical approval and written consent were obtained. The samples were obtained, processed, and stored following the Human Tissue Act 2004. The confidentiality and anonymity of the participants were strictly maintained throughout the study, with samples only identified by study ID number and all associated data anonymized. Clinical measurements of diabetes duration, blood pressure, HbA1c, estimated glomerular filtration rate (eGFR), height, weight, and urinary albumin/creatinine ratio were obtained.

### 2.9. RNA Extraction From Blood and Urine

*Patient blood samples:* Blood samples (approximately 10 mL) were collected in EDTA‐coated tubes and centrifuged for 10 min at 200× g no more than 4 h after collection. The leukocyte interphase (buffy coat) was collected and placed into TRIzol (ThermoFisher Scientific) before being stored at −80°C for future analysis.

*Patient urine samples:* Patients performed an overnight urine collection at home. The next day, we obtained the urine sample (volumes ranged from 200 to 3000 mL), which was centrifuged at 2500× g for 20 min. Urinary sediment (containing the RNA) was resuspended in TRIzol before being stored at −80°C for future analysis.

*RNA extraction:* RNA extraction was performed using the phenol/chloroform method. cDNA synthesis and RT‐PCR for *BCL-X* splice isoforms were performed as described above.

### 2.10. Statistical Analysis

The data analysis was performed in GraphPad Prism (Prism 10). Unpaired student *t*‐test (parametric) or Mann–Whitney *U* test (nonparametric tests) was used to compare a two‐group data set, and for three or more groups of data sets, a one‐way analysis of variance (ANOVA) parametric or Kruskal–Wallis (nonparametric tests) was used. To assess the linear relationship between two sets of data, a Pearson or Spearman correlation test (depending on the distribution of the data) was performed following the normality test. All analyses performed were only considered statistically significant if the *p* value was less than 0.05.

## 3. Results

### 3.1. Rnaseq Analysis of AS Events Identifies *BCL-X* Splice Variants to Be Associated With DN

With the goal to discover splice variants associated with the progression of DN, we performed RNAseq analysis on GEnCs. To mimic the diabetic environment, GEnCs were exposed to the GS (25 mM glucose, 1 ng/mL TNF‐*α*, 1 ng/mL IL‐6, and 100 nM insulin) for 1 week. NG (5.5 mM glucose) and MAN (5.5 mM glucose and 19.5 mM mannitol, to account for osmotic properties) were used as controls. RNA was extracted, short‐reads RNAseq analysis was performed, and data were analyzed with DESeq2 for gene expression and MISO for differential splice variants.

In the DESeq2 differential expression analysis, using a cutoff of log2 fold change of 0.5 and *q* < 0.05, 124 genes were found to be significantly upregulated and 33 genes significantly downregulated in GEnCs exposed to the GS for 1 week, in comparison with the MAN control (Figure 1a). Gene ontology (GO) enrichment using the GOrilla tool identified eight GO terms that were significantly enriched (Figure 1b). In the heat maps of the two GO terms with the highest enrichment scores, “chemokine receptor binding” and “inflammatory response”, several genes are shown that are known to be involved in the pathogenesis of diabetes (for example, *CXCL10, IL-6,* and *NFKB1*) (Figure 1c). A set of 11 genes (six upregulated and five downregulated) were validated using qRT‐PCR; nine out of 11 (82%) showed changes in expression as predicted by the bioinformatics analysis (Figure 1d).

Figure 1DESeq2 analysis of gene expression changes in glomerular endothelial cells (GEnCs) exposed to a diabetic environment. Differential expression analysis using the DESeq2 Bioconductor package. (a) In total, 124 genes were found to be significantly upregulated, and 33 genes significantly downregulated in GEnCs exposed to the glucose soup (GS) for 1 week, in comparison to the mannitol (MAN) control (*n* = 5). (b) Gene ontology (GO) enrichment using the GOrilla tool identified eight GO terms that were enriched. (c) Heat maps of the two GO terms with the highest enrichment scores, “chemokine receptor binding” and “inflammatory response”. (d) Out of 11 genes found to be dysregulated (six upregulated and five downregulated), nine were validated using qRT‐PCR (82%) (*n* = 3–12 biological repeats;  ^∗^ *p* < 0.05 versus MAN control as assessed by Student′s *t*‐test).

**Figure:**
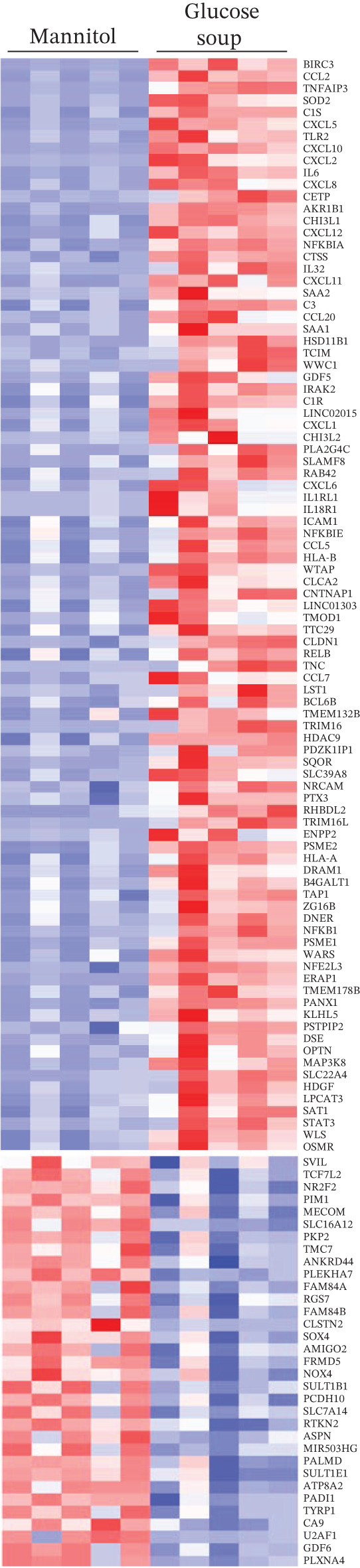
(a)

**Figure:**
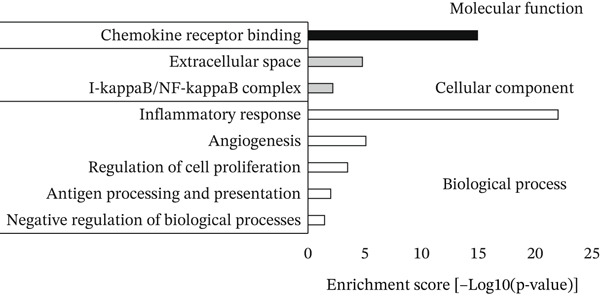
(b)

**Figure:**
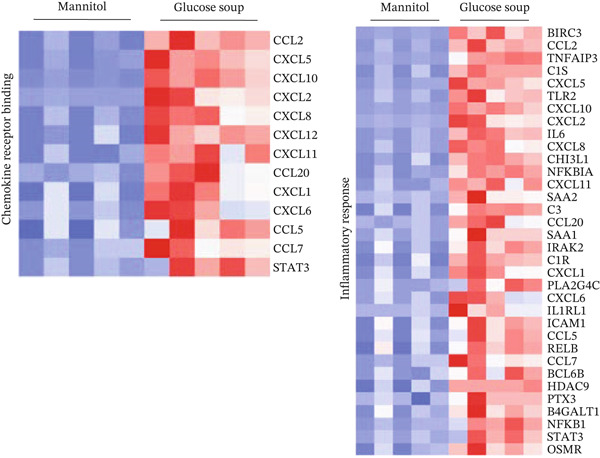
(c)

**Figure:**
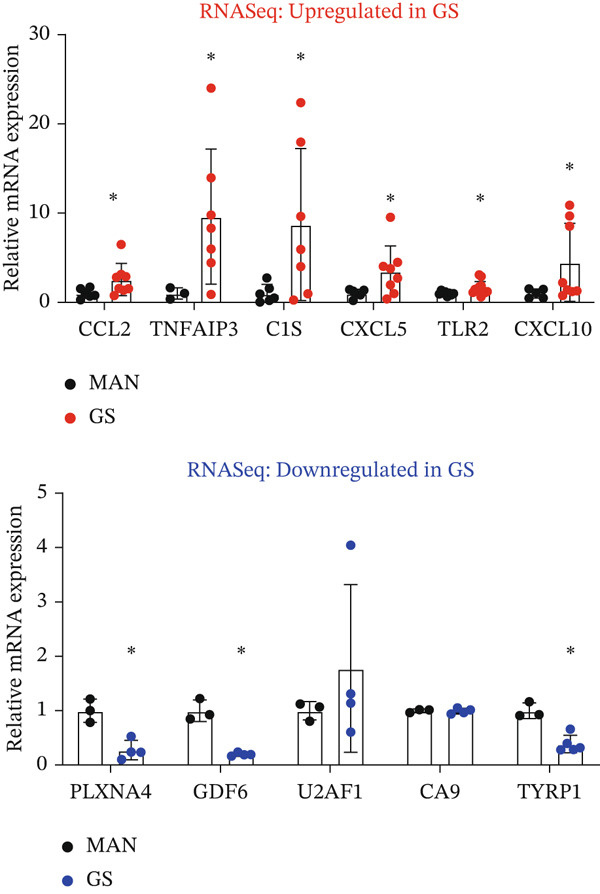
(d)

MISO is a probabilistic framework used to quantify the expression level of AS genes from RNAseq data. We identified 103 significant AS events: five skipped exon (SE), 12 alternative 5^′^ splice site (A5SS), 11 alternative 3^′^ splice site (A3SS), 26 mutually exclusive exon (MXE), and 39 retained intron (RI) events (Figure 2a). We implemented a *Δ*PSI cutoff of 0.2 and Bayes factor cutoff of 10 to select AS events that have changed significantly between conditions. All protein‐coding events were validated using RT‐PCR and 70% were found to be as predicted by the bioinformatics analysis. Several of the AS events that change upon GS incubation are presented in Table 1. Example RT‐PCRs of four types of events and the way they change upon GS incubation (*ZNF426, DOK1, ARAP1,* and *BCL-X*) are shown in Figure 2b.

Figure 2MISO analysis of significant alternative splicing events in glomerular endothelial cells (GEnCs) exposed to glucose soup (GS). MISO (mixture‐of‐isoforms) is a probabilistic framework used to quantify the expression level of alternatively spliced genes from RNAseq data. (a) Using the MISO framework, we identified 103 significant alternative splicing (AS) events (five skipped exon (SE), 12 alternative 5^′^ splice site (A5SS), 11 alternative 3^′^ splice site (A3SS), 36 mutually exclusive exon (MXE), and 39 retained intron (RI) events). We implemented a *Δ*PSI cutoff of 0.2 and Bayes factor cut‐off of 10 to select significantly (AS) events (*n* = 5). (b) All events were validated using RT‐PCR; an example of four types of events (*ZNF426, DOK1, ARAP1,* and *BCL-X*) are shown (*n* = 3–8 biological repeats;  ^∗^ *p* < 0.05 versus mannitol (MAN) control as assessed by Student′s *t*‐test). We were able to validate 70% of protein‐coding AS events.

**Figure:**
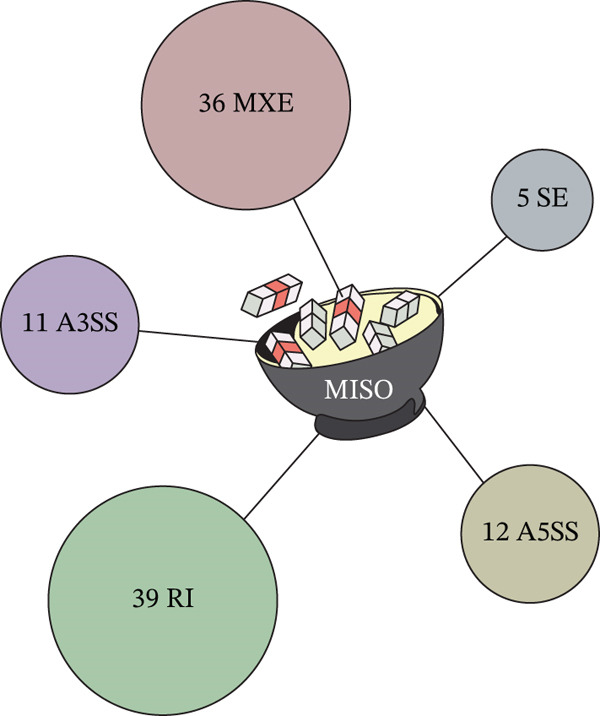
(a)

**Figure:**
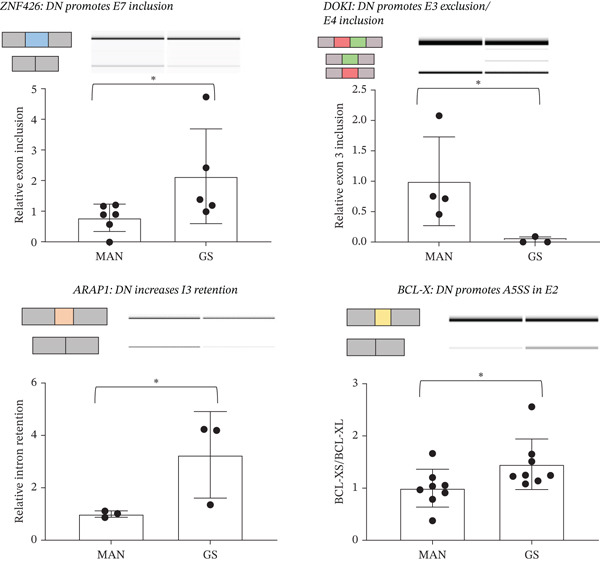
(b)

**Table 1:** MISO analysis of interesting alternative splicing events significantly dysregulated in GEnCs exposed to diabetic conditions (glucose soup) in comparison with a normal glucose and osmotic control ^∗^.

Gene	Event	Description	Protein	Validated with RT‐PCR
*DOK1*	MXE	Scaffold protein. Negative regulator of insulin‐signaling pathway	Truncated C‐terminus missing essential tyrosine docking sites	*p* < 0.05
*IRAK1*	MXE	IL‐1 receptor associated kinase—we see an increase in the 1b isoform	Lacks an internal segment—IRAK1b does not undergo modification or autophosphorylation and is resistant to degradation	*p* < 0.05
*MDM2*	MXE	Proto‐oncogene—nuclear localized E3 ubiquitin ligase.	Isoform lacks nuclear localization and export sequences	*p* < 0.05
*TRA2A*	MXE	Regulates pre‐mRNA splicing	Unknown effect	—
*ARAP1*	IR	Rho GTPase and GPCR signaling—kidney hypertrophy and apoptosis	Unknown effect	*p* < 0.05
*GSTK1*	IR	Enzyme required for cellular detoxification	Shorter isoform—lacks internal segment	—
*MCRIP1*	IR	MAP kinase‐signaling pathway involved in epithelial‐mesenchymal transition	Shorter isoform may alter ERK signaling	p <0.05
*BCL-X*	A5SS	Apoptosis	Increase in the apoptotic Bcl‐xS	p < 0.05

Abbreviations: A3SS, alternative 3^′^ splice site; A5SS, alternative 5^′^ splice site; IR, intron retention; MXE, mutually exclusive exons; SE, skipped exon.

^∗^Excluding nonprotein coding genes.

The list of splicing events that move when exposed to a diabetic environment (GS), as described in Table 1, included a switch in *BCL-X* isoforms. *BCL-X* has two splice isoforms, *BCL-XS* and *BCL-XL,* which result from a 5^′^ AS event (Figure 3a) and have opposing functions on apoptosis [18]. The functional importance of this splicing switch has been studied extensively in cancer [19]; therefore, we wanted to explore its involvement in the pathogenesis of DN.

Figure 3Glomerular endothelial cells (GEnCs) exposed to a diabetic environment have an upregulation of *BCL-XS/BCL-XL*, resulting in apoptosis. (a) Diagrammatic representation of the *BCL-X* alternative splicing (AS) event. Use of an alternative 5^′^ splice site in exon 2 of the *BCL-X* pre‐mRNA results in the expression of the proapoptotic *BCL-XS* isoform, in contrast to the antiapoptotic *BCL-XL*. (b) The switch in *BCL-X* AS to promote BCL‐XS expression in response to treatment of GEnCs with glucose soup (GS) was confirmed at the protein level using Western blotting (*n* = 6 biological repeats;  ^∗^ *p* < 0.05 versus mannitol (MAN) and normal glucose (NG) controls, as assessed by one‐way ANOVA and Tukey post‐hoc test). (c) Using the JC‐10 assay, GEnCs treated with GS showed an increase in the fluorescence green/red (520/590 nm) ratio, that is, mitochondrial depolarization, representing an increase in cell apoptosis, compared with MAN and NG controls (*n* = 4 biological repeats;  ^∗^ *p* < 0.05 versus NG and MAN as assessed by one‐way ANOVA and Tukey post‐hoc test). (d) GEnCs treated with GS also had a significantly reduction in viability assessed with the trypan blue assay (*n* = 3 biological repeats;  ^∗^ *p* < 0.05 versus MAN and normal glucose (NG) controls as assessed by one‐way ANOVA and Tukey post‐hoc test). (e) HEK293 cells exposed to GS for 24 h were transfected with a control plasmid (CP) or BCL‐XL overexpressing plasmid (P). The JC‐10 apoptosis assay was performed 48 h after treatment, which showed that overexpression of BCL‐XL decreased mitochondrial membrane polarization, and therefore apoptosis, compared with the GS and GS + CP controls (*n* = 3 biological repeats;  ^∗∗^ *p* < 0.01 versus GS and GS + CP as assessed by one‐way ANOVA and Tukey post‐hoc test).

**Figure:**
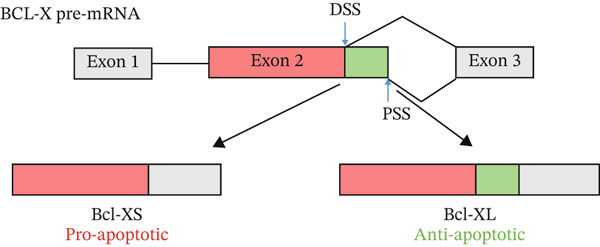
(a)

**Figure:**
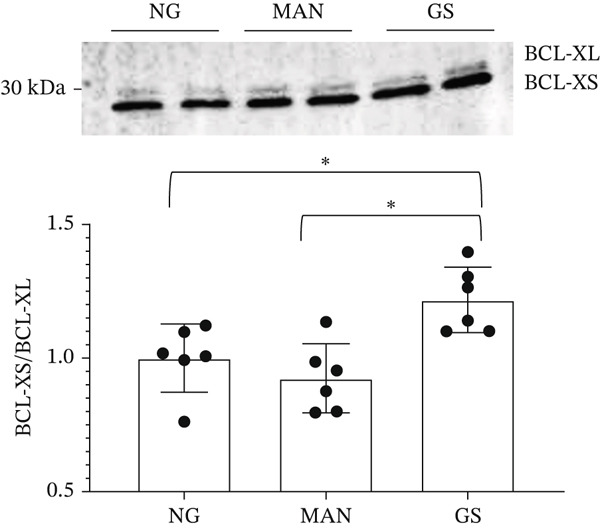
(b)

**Figure:**
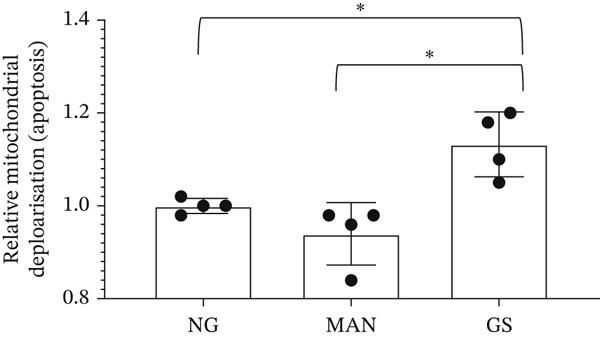
(c)

**Figure:**
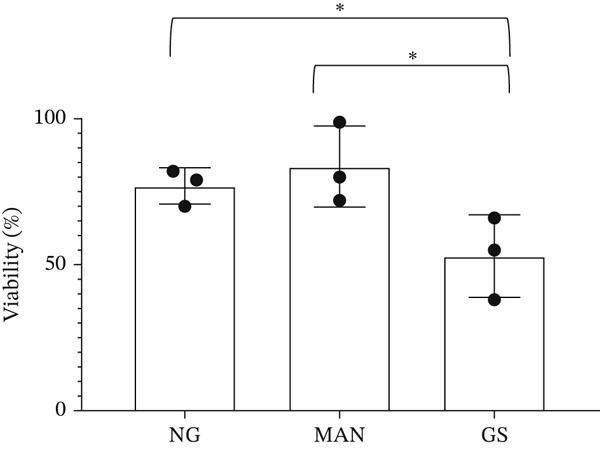
(d)

**Figure:**
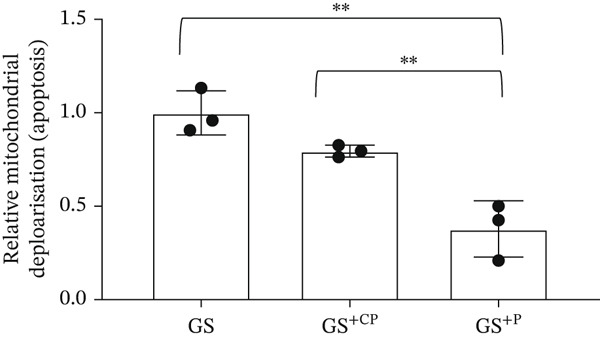
(e)

### 3.2. The Diabetic Environment (GS) Increases the *BCL-XS/BCL-XL* Ratio in GEnCs and HEK293 Cells, Increasing Apoptosis

The analysis of *BCL-X* AS described above was performed at the RNA level; however, the ultimate effectors of cellular function are proteins. Therefore, we investigated the ratio of BCL‐X protein isoforms. As observed in the western blotting analysis in Figure 3b, upon exposure of GEnCs to GS, the ratio of BCL‐XS to BCL‐XL is increased. The same ratio does not increase upon exposure to MAN. Furthermore, we confirmed that this switch in isoforms functionally resulted in an increase in apoptosis and cell death, as measured using the JC‐10 and Trypan Blue assays (Figure 3c,d).

We wanted to further explore the effects of a diabetic environment on the *BCL-X* splicing switch and cell apoptosis. Therefore, we tested other conditions, including incubation of cells with only HG instead of GS, in addition to changes in the exposure mode, such as oscillating high‐glucose (OHG)/oscillating glucose soup (OGS). Oscillating treatment involves changing the media from NG to either MAN or HG back and forth every 12 h, for 48 h. As observed in Figures S1, S2, S3, and S4, OHG, GS, and OGS, but not HG, increased the ratio of BCL‐XS to BCL‐XL, as well as apoptosis, in HEK293 cells.

### 3.3. Overexpression of the *BCL-XL* Isoform Rescues the Apoptosis Induced by the Diabetic Environment

There are many determinants of apoptosis that are active during the progression of DN. The *BCL-X* splicing switch might be a consequence of the DN environment. Therefore, the functional importance of this splicing switch needs to be investigated, that is, if we switch back the isoform ratio to that of the physiologically normal situation by increasing the expression of antiapoptotic *BCL-XL*, can we see a rescue, at least partially, of apoptosis?

To address this question, we used plasmid‐induced overexpression of the antiapoptotic *BCL-XL* isoform (see Figure S5 for proof of expression). HEK293 cells, with or without *BCL-XL* overexpression, were exposed to NG, MAN, and GS. As seen in Figure 3e, we observed a significant decrease in mitochondrial depolarization induced by GS exposure in cells transfected with the *BCL-XL* plasmid using the JC‐10 assay, suggesting that BCL‐XL overexpression decreased GS‐induced apoptosis in HEK293 cells.

### 3.4. The *BCL-XS/BCL-XL* Ratio Correlates With Various Clinical Parameters Measured in the Blood and/or Urine of Patients With DN

The in vitro studies showed that the *BCL-X* splicing isoform ratio changes in renal cells exposed to a diabetic environment result in an increase in the *BCL-XS* isoform and, subsequently, an increase in cellular apoptosis. Moreover, overexpression of the *BCL-XL* isoform can partially rescue the apoptosis. This suggests that the *BCL-X* splicing switch is important in the development of the DN. Therefore, we investigated whether the *BCL-X* isoform ratio is associated with clinical parameters of the progression of DN.

We analyzed 45 blood samples and 28 urine samples from healthy and diabetic patients with varying stages of nephropathy. Patients were enrolled in the BEAt‐DKD study [20], an EU IMI funded study which is a partnership between public and private sectors that aims to improve prevention and management of diabetic kidney disease, and the VIBE study (see acknowledgements), including patients with Type 2 diabetes (see Table 2). RNA was extracted from the urinary sediment or leukocytes, RT‐PCR was performed for the *BCL-X* splice isoforms (see an example in Figure S6), and the ratio of isoforms was found to correlate with various clinical parameters, as described below.

**Table 2:** Patient characteristics.

Characteristics			
	Control	Normoalbuminuric	Microalbuminuric
*N*	27	36	18
Age years	70.1 ± 7.3	68.4 ± 6.5	73.6 ± 5.2
Gender			
Male	16 (19.8)	20 (24.7)	15 (18.5)
Female	11 (13.6)	16 (19.6)	y (3,7)
BMI	29.7 ± 5.4	29.3 ± 4.4	27.0 ± 0.4
HbA1c	41.5 ± 7.3	58.0 ± 15.5	62.2 ± 10.2
Blood glucose	5.6 ± 1.3	8.1 ± 2.6	8.6 ± 1.9
SBP	131.6 ± 9.6	133.5 ± 14.5	136.4 ± 29.1
DBP	80.9 ± 11.77	82.6 ± 14.2	77.8 ± 11.8

*Note:* Data are presented as *n (%)* or *mean (±SD).* Percentages were calculated based on the total number of patients.

Abbreviations: BMI, body mass index; DBP, diastolic blood pressure; HbA1c, glycated hemoglobin; n, sample number; N, total number of patients; SBP, systolic blood pressure.

A negative correlation was observed between the (eGFR) and the *BCL-XS/BCL-XL* ratio from urine sediments (Pearson *r* = –0.3934, *p* = 0.0468); however, the eGFR did not correlate with the *BCL-XS/BCL-XL* ratio in leucocytes (Spearman *r* = –0.04143, *p* = 0.972) (Figure 4a,b). The urinary albumin creatinine ratio (UACR) correlated positively with the leucocyte *BCL-XS/BCL-XL* ratio (Pearson *r* = 0.4522, *p* = 0.0038), but it did not correlate with *BCL-XS/BCL-XL* ratio in urinary sediment (Spearman *r* = –0.05321, *p* = 0.8141) (Figure 4c,d). Microalbuminuric patients did not show a change in the *BCL-XS/BCL-XL* expression ratio in leukocytes; however, the *BCL-XS/BCL-XL* expression ratio obtained from urine showed an increase in microalbuminuric patients compared with controls; however, as we only had data for two patients, we could not perform statistical analysis (Figure 4e,f). Other parameters, including serum creatinine, Hb1Ac, fasting glucose, and gender, did not correlate with the ratio of *BCL-X* isoforms (Figures S7, S8, S9, and S10). The fact that the *BCL-X* isoform ratio correlated with some of the parameters of DN progression further strengthens the conclusion from the in vitro studies that this splicing event is important in development of DN.

Figure 4The *BCL-X* isoform ratio correlates with some clinical parameters in diabetic nephropathy patients. (a) The *BCL-XS/BCL-XL* ratio obtained from urine sediment has a negative correlation with the estimated glomerular filtration rate (eGFR): cDNA was generated from RNA extracted from sediments of urine collected from patients overnight. RT‐PCR was performed using *BCL-X* primers to amplify the XL and XS isoforms. The bioanalyser was used to quantify the isoform concentration and the *BCL-XS/BCL-XL* ratio was calculated. A plot of eGFR against the urinary sediment *BCL-XS/BCL-XL* ratio was generated using GraphPad prism. The data was analyzed using Pearson correlation analysis (*r* = 0.3934, *p* = 0.0468, *n* = 26 overnight urine samples). (b) The *BCL-XS/BCL-XL* ratio obtained from leucocytes does not correlate with the eGFR: cDNA was generated from RNA extracted from leucocytes. A Spearman correlation analysis was performed (*r* = −0.04143, *p* = 0.7920, *n* = 43 blood samples). (c) The *BCL-XS/BCL-XL* ratio from urine sediment does not correlate with the urine albumin creatinine ratio (uACR) (Spearman *r* = –0.05321, ns *p* = 0.9187, *n* = 22 samples). (d) The *BCL-XS/BCL-XL* ratio from leucocytes has a positive correlation with the uACR in participants without clinically significant albuminuria (Pearson *r* = 0.4522, *p* = 0.0361, *n* = 39 blood samples). (e) The *BCL-XS/BCL-XL* ratio obtained from urine sediment is not significantly increased in normoalbuminuria (*n* = 11) compared with controls (*n* = 15; p = ns as measured with the Student′s *t*‐test). Although an increase was observed in microalbuminuria, only two patients met the criteria, and statistical analysis could not be performed. (f) The leucocyte *BCL-XS/BCL-XL* ratio is not significantly increased in normoalbuminuria (*n* = 24) or microalbuminuria (*n* = 15) compared with controls (*n* = 5). The data was statistically analyzed using the Mann–Whitney test.

**Figure:**
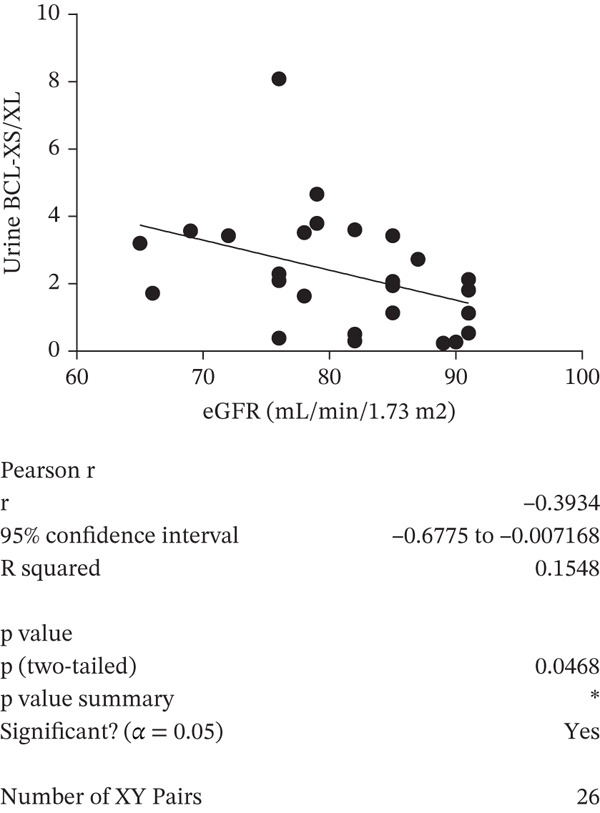
(a)

**Figure:**
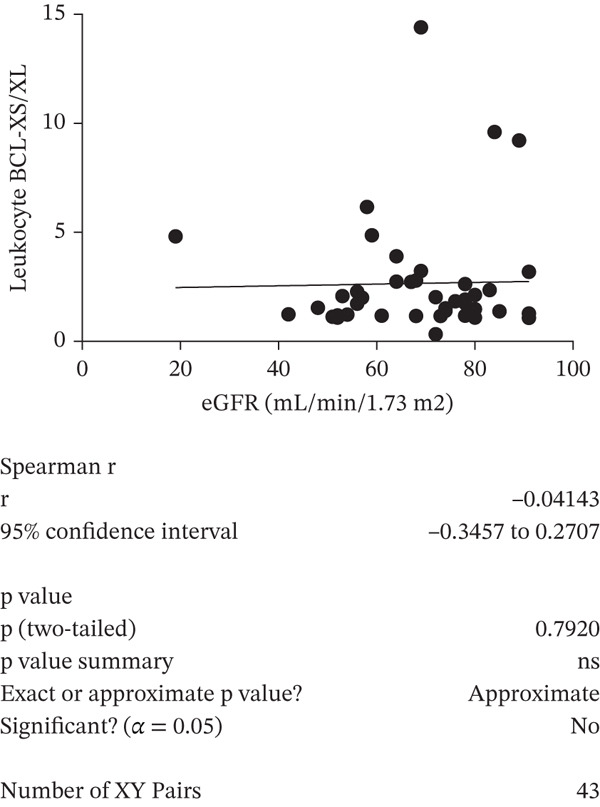
(b)

**Figure:**
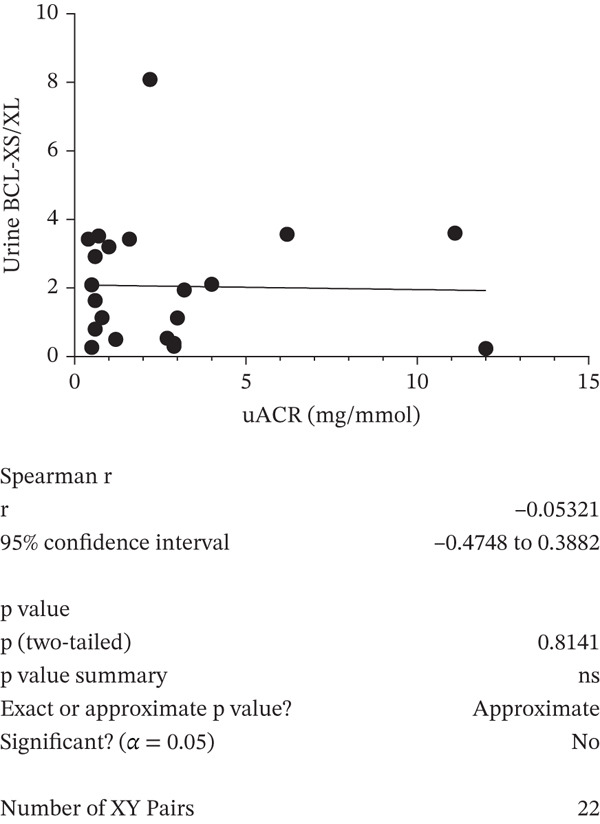
(c)

**Figure:**
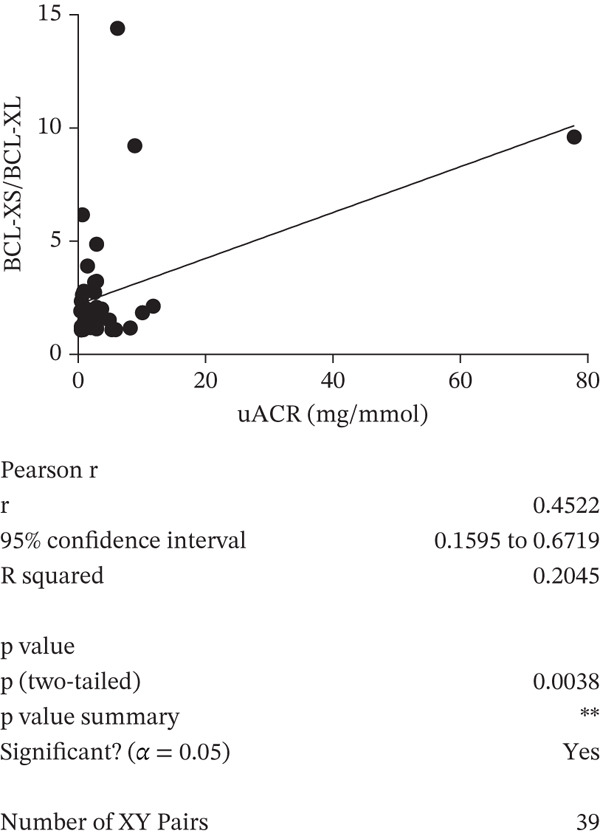
(d)

**Figure:**
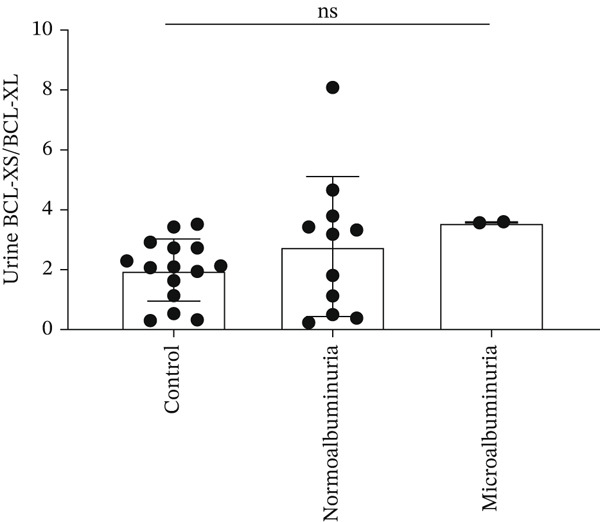
(e)

**Figure:**
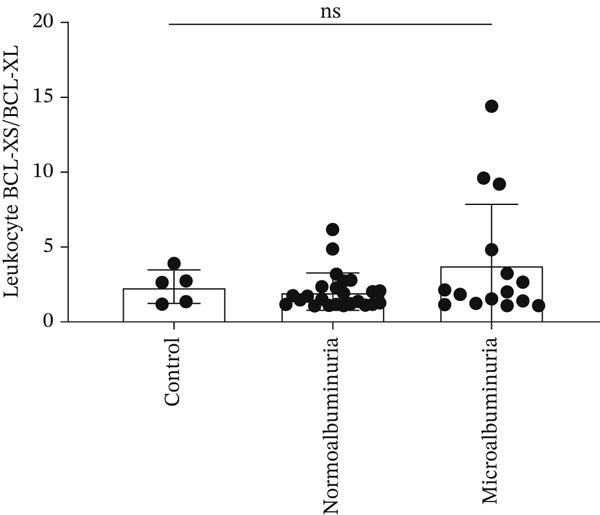
(f)

### 3.5. An Increase in *IL-6* Regulates *BCL-X* Splicing to Promote *BCL-XS* Expression in Diabetic GEnCs

The experiment described above highlighted the importance of *BCL-X* splicing in DN; therefore, we explored which molecules are important for its regulation in this context

IL‐6 has previously been reported to repress *BCL-XL* expression through an intron retention element (IRE) in intron 2 of the *BCL-X* pre‐mRNA [18], although the exact mechanism is unknown (Figure 5a). Analysis of RNAseq data described above showed that *IL-6* expression is increased in GEnCs exposed to a diabetic environment (GS treatment) (Figure 5b). Indeed, qRT‐PCR validation showed a clear increase in *IL-6* expression upon exposure to GEnCs. The GS contains *IL-6*; however, treating cells with GS that did not contain IL‐6 showed a similar increase in IL‐6 expression (Figure 5c). Treatment of GEnCs with IL‐6 resulted in a dose‐dependent increase in the ratio of *BCL-XS* to *BCL-XL* (Figure 5d), in addition to a corresponding increase in apoptosis as ascertained by JC‐10 and caspase activation assays (Figure 5e,f), and a decrease in viability shown with the trypan blue assay (Figure 5g). Furthermore, exposure of HEK293 cells to increasing concentrations of IL‐6 resulted in a dose‐dependent increase in the ratio of *BCL-XS* to *BCL-XL* and a corresponding increase in apoptosis (Figure S11).

Figure 5IL‐6 is a major regulator of *BCL-X* splicing. (a) Schematic of *BCL-X* exon 2 and position of the intron response element (IRE). (b) RNAseq and DESeq2 analysis of glomerular endothelial cells (GEnCs) treated with glucose soup (GS) showed a significant increase in the IL‐6 read counts compared with mannitol (MAN) control (*n* = 5;  ^∗^ *p* < 0.05). (c) The increase in IL‐6 expression in response to GS was confirmed with qRT‐PCR, which persisted in the absence of IL‐6 from the GS treatment (*n* = 3;  ^∗^ *p* < 0.05 versus. MAN control as assessed by one‐way ANOVA and Tukey post‐hoc test). (d) Treatment of GEnCs for 6 h with increasing concentrations of IL‐6 alone resulted in a dose‐dependent increase in *BCL-XS/BCL-XL* (*n* = 3;  ^∗^ *p* < 0.05 as assessed by Kruskall–Wallis). (e) Using the JC‐10 assay, GEnCs treated with IL‐6 showed a dose‐dependent increase in the fluorescence green/red (520/590 nm) ratio, that is, mitochondrial depolarization, representing an increase in cell apoptosis, compared with MAN control (*n* = 3–4;  ^∗^ *p* < 0.05 versus 0 ng/ml IL‐6 as assessed by one‐way ANOVA and Tukey post‐hoc test). (f) IL‐6 dose‐dependently increased caspase activation, indicating increased cell apoptosis (*n* = 3;  ^∗^ *p* < 0.05 versus 0 ng/ml IL‐6 as assessed by one‐way ANOVA and Tukey post‐hoc test). (g) IL‐6 dose‐dependently decreased cell viability, as assessed with trypan blue staining, indicating increased cell death (*n* = 3;  ^∗^ *p* < 0.05 as assessed by one‐way ANOVA).

**Figure:**
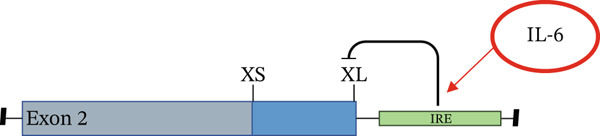
(a)

**Figure:**
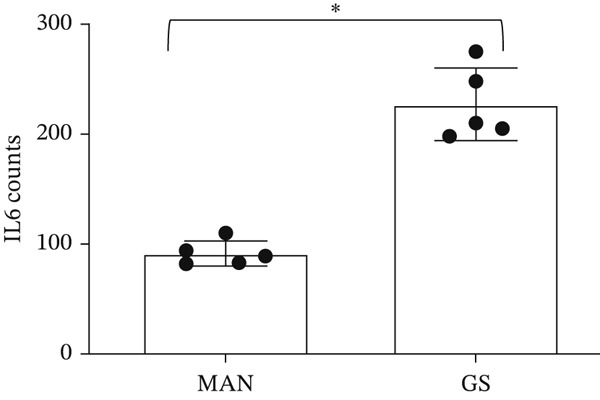
(b)

**Figure:**
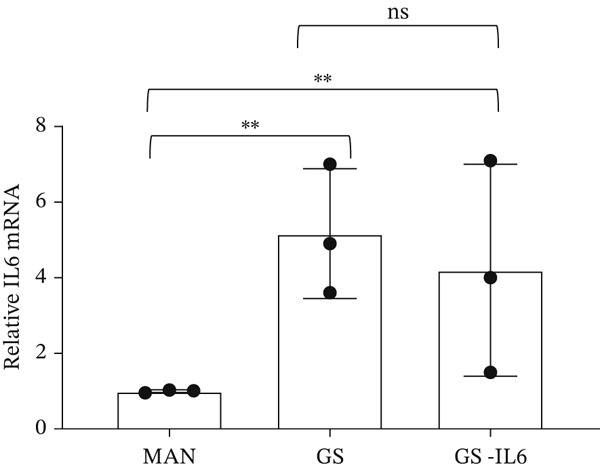
(c)

**Figure:**
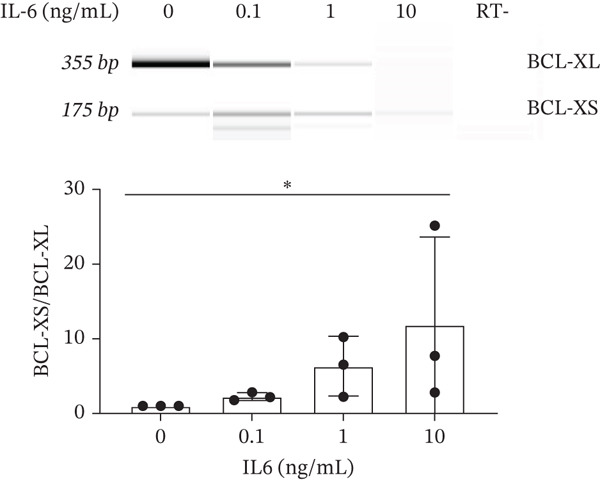
(d)

**Figure:**
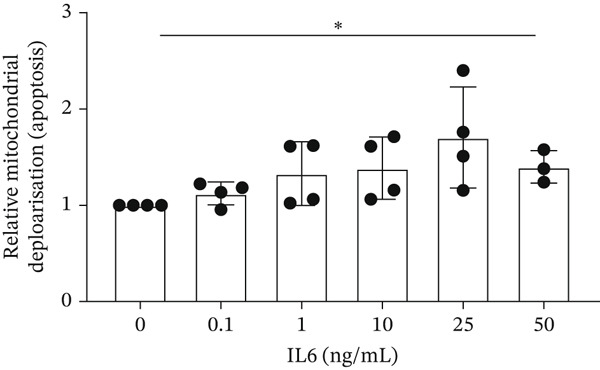
(e)

**Figure:**
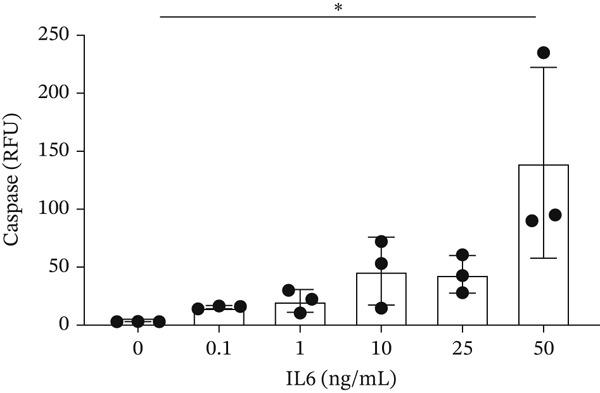
(f)

**Figure:**
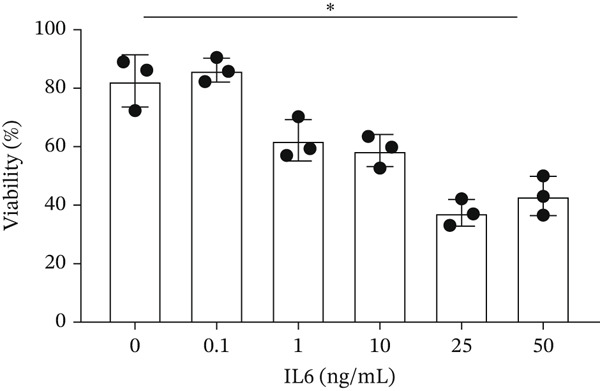
(g)

### 3.6. Expression of Splice Factors *SF3B1* and *PTBP1* Is Decreased in a Diabetic Environment, Which May Regulate the *BCL-X* Splicing Switch

We further investigated which splice factors may be involved in the regulation of *BCL-X* splicing in the diabetic context.

SF3B1 has previously been reported to repress *BCL-XS* splice site selection through binding to ceramide‐responsive RNA cis‐element 1 (CRCE1) in exon 2 of the *BCL-X* pre‐mRNA [21] (Figure 6a). RNAseq and DESeq2 analysis of GEnCs treated with GS showed a significant decrease in the *SF3B1* read counts compared with MAN control; this was validated by qRT‐PCR (Figure 6b). The downregulation of SF3B1 when GEnCs are incubated in GS was confirmed at the protein level with immunofluorescence (Figure 6c). Interestingly, the expression of SF3B1 is not regulated by IL‐6 (Figure S12).

Figure 6The splice factors *SF3B1* and *PTBP1* are downregulated in response to glucose soup (GS) treatment of glomerular endothelial cells (GEnCs). (a) *SF3B1* binds to a ceramide‐responsive RNA cis‐element 1 (CRCE1) in exon 2 of the *BCL-X* pre‐mRNA and represses *BCL-XS*. (b) qRT‐PCR shows a decrease in *SF3B1* expression when cells are incubated in GS (*n* = 6–8;  ^∗^p < 0.05 versus mannitol (MAN) control, as assessed by the Student′s *t*‐test). (c) Quantitation of immunofluorescence for SF3B1 confirms the decrease at the protein level (*n* = 4;  ^∗^ *p* < 0.05 versus MAN control, as assessed by the Student′s *t*‐test). (d) qRT‐PCR shows a decrease in *PTBP1* expression when cells are incubated in GS (*n* = 5;  ^∗^ *p* < 0.05 versus MAN control, as assessed by the Student′s *t*‐test). (e) Urinary *PTBP1* expression correlated with the estimated glomerular filtration rate (eGFR) and serum creatinine (SCr) in the urine samples from diabetic patients (eGFR: Spearman *r* = 0.5203, *p* = 0.0408, *n* = 16; SCr: Spearman *r* = –0.6401, *p* = 0.009, *n* = 16).

**Figure:**
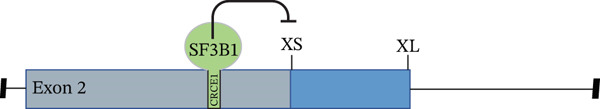
(a)

**Figure:**
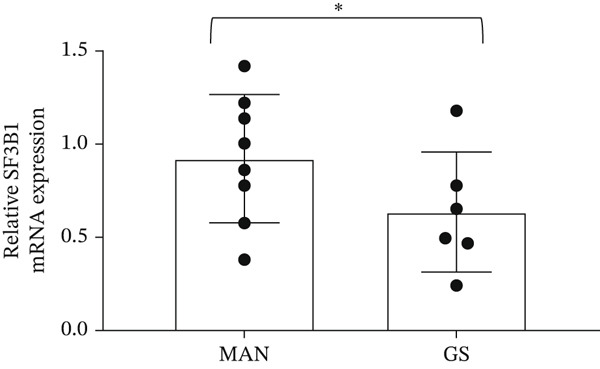
(b)

**Figure:**
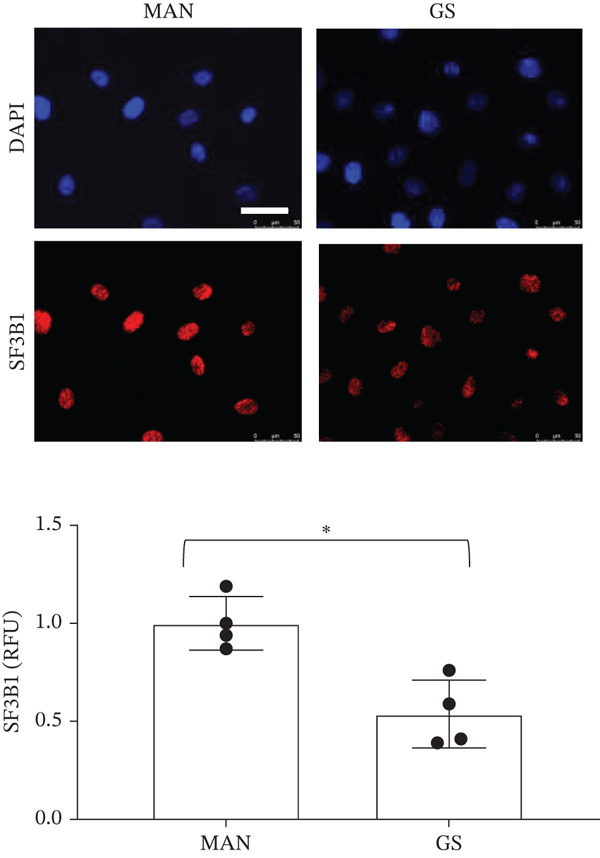
(c)

**Figure:**
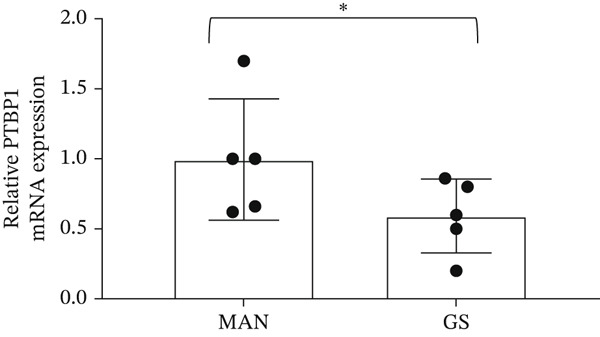
(d)

**Figure:**
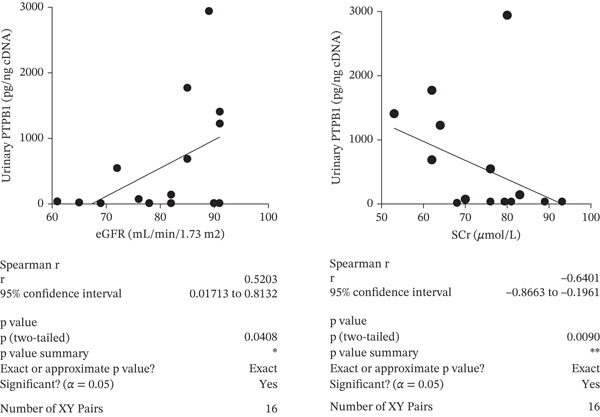
(e)

Another splice factor reported to regulate *BCL-X* splicing, *PTBP1*, is decreased when GEnCs are incubated in GS (qRT‐PCR) (Figure 6d). Interestingly, the urinary *PTBP1* expression in patients with various degrees of DN correlated with markers of a decline in renal function, that is, a positive correlation with the eGFR (Spearman *r* = 0.5203; *p* = 0.0408) and a negative correlation with serum creatinine (Spearman *r* = –0.6401; *p* = 0.009) (Figure 6e).

## 4. Discussion and Conclusions

AS is a major level of gene regulation that is able to drive various cellular functions in that are sometimes autonomously from transcription. Splicing variants are described in virtually every class of molecules, from growth factors to tyrosine kinase/phosphatase receptors to tumor suppressors and oncogenes. These splicing isoforms often have opposing functions, for example, pro‐ or antiangiogenic, pro‐ or antiapoptotic [11, 22]. There is recent evidence that the occurrence of a particular set of splice isoforms is a determinant for both physiological and pathological processes [23]. Indeed, there are several examples were various biological processes are coordinated by networks of AS regulators, for example, tissue and organ development [24, 25], cholesterol biosynthesis and uptake [26], pancreatic beta cells function and survival [27], or epithelial‐mesenchymal transitions [28]. Interestingly, the function of a large number of apoptosis genes is controlled by AS [29, 30]. Thus, it is highly likely that this may occur in other processes, including DN, and may represent a novel paradigm in biology.

Diabetes is characterized by hyperglycemia and the activation of proinflammatory cytokines. Hyperglycemia has been shown to cause programmed cell death in a variety of renal cells, including endothelial and podocyte cells, which have been implicated in the pathogenesis of DN [31, 32]. Therefore, understanding the mechanism involved in the antiapoptotic to proapoptotic splicing in *BCL-X* (a major apoptosis gene) in renal cells may be a key to rescuing renal cells from apoptosis and could also serve as a novel biomarker for DN severity.

This study is aimed at determining whether AS events identified in DN can be targeted as a therapeutic strategy, as well as whether they are associated with DN severity.

We discovered that the *BCL-X* AS event is dysregulated in diabetic GEnCs using RNAseq analysis. Recognizing the associated programmed cell death that comes with DN, we sought to further understand the mechanism of *BCL-X* AS in diabetic conditions and how it can be exploited to rescue diabetic cells from apoptosis, as well as its potential as a biomarker.

A systemic increase in IL‐6, together with other cytokines, has been associated with obesity‐related inflammation. This increase has also been implicated as a risk factor in the development of insulin resistance and Type 2 diabetes [33, 34]. Studies have shown that IL‐6 regulates *BCL-X* AS in K562 cells in favor of the proapoptotic *BCL-XS* isoform [35]. RT‐PCR for the *BCL-X* isoforms showed an increase in *BCL-XS* band intensity as the IL‐6 concentration increased from 0.1 ng/mL to 50 ng/mL. Moreover, the JC‐10 assay showed an increase in apoptosis of GEnCs and HEK293 cells as the IL‐6 concentration increased. These findings indicate that *BCL-X* AS may be dependent on IL‐6 in a dose‐wise manner.

Hyperglycemia is a hallmark of diabetes and has also been associated with increased IL‐6 levels [36]. Consequently, it was necessary to determine the impact of a HG environment on *BCL-X* AS. GS treatment caused a significant increase in the *BCL-XS/BCL-XL* ratio compared with NG in HEK293 cells, in addition to a significant increase in apoptosis.

Additionally, studies have demonstrated that OHG could be more deleterious on endothelial function in comparison with stable or continuous HG, and can promote the hyperactivation of p53, a molecule known to be involved in apoptosis [37]. Investigating the effects of OHG on *BCL-X* AS showed that OHG treatment caused a significant increase in the *BCL-XS/BCL-XL* ratio compared with NG, with a corresponding increase in apoptosis in HEK293 cells. Furthermore, exposure to OGS+ showed a significant increase in *BCL-XS/BCL-XL* ratio compared with NG, with a corresponding increase in apoptosis. We therefore concluded that IL‐6, or a combination of OHG and IL‐6, plays a key role in *BCL-X* AS, which consequently leads to apoptosis.

As a proof of principle, we saw a significant decrease in apoptosis in HEK293 cells overexpressing *BCL-XL* following exposure to GS compared with cells transfected with a control. This preliminary work serves as evidence that regulation of *BCL-X* AS to promote *BCL-XL* expression could be a potential therapeutic strategy in diabetic renal cells.

Using Pearson correlation analysis, we attempted to establish a relationship between kidney function parameters in research participants and the urine sediment or leucocyte *BCL-XS/BCL-XL* ratio. The kidney function metric eGFR was discovered to weakly correlate with the *BCL-XS/BCL-XL* ratio acquired from patients′ urine sediments, whereas uACR moderately correlated with the *BCL-XS/BCL-XL* ratio obtained from leucocytes from patient blood. It is important to note that, although serum creatinine is used to assess renal function, additional confounding factors influence creatinine production and secretion. Muscle mass is a factor that influences creatine synthesis, levels, and, consequently, creatinine production. Age and gender appear to be the most important factors in influencing total muscle mass and, thus, creatinine production. Men create more creatinine than women because they have larger muscular mass. Lower creatinine production can also result from age‐related muscle mass reduction [38]. Furthermore, the amount of dietary creatine taken, typically in the form of meat, influences creatinine synthesis as well as total muscle mass [39]. Reduced creatinine synthesis is associated with pathophysiologic diseases that cause muscle atrophy and decrease muscle mass. These circumstances include long‐term glucocorticoid medication, muscular dystrophy, and paralysis just to mention a few. Since multiple unconnected factors may have simultaneous effects on serum creatinine levels, it is not ideal for renal function assessment. However, it is much more advantageous to use serum creatinine, due to its low cost and availability. These drawbacks in serum creatinine levels estimation could be the reason why the *BCL-XS/BCL-XL* ratio from leucocyte and urine sediment did not correlate with serum creatinine.

Despite the limits and inconveniences of measuring albuminuria, chronic disease markers have been employed for many years. However, in the lack of other known and tested approaches, they are used continuously with corrections and considerations, making them more onerous. Other urine markers mentioned in reviews include Neutrophil gelatinase‐associated lipocalin (NGAL), N‐acetyl‐beta‐glucosaminidase (NAG), Type IV collagen, and nephrin, to name a few (40). These markers are expected to be more durable than those now available. Future research could investigate the link between these new indicators and *BCL-X* AS. Furthermore, a larger cohort will solidify our findings.

In conclusion, we discovered that OHG and IL‐6 impact *BCL-X* AS, increasing the proapoptotic isoform *BCL-XS* and inducing cell apoptosis. We further propose that increasing the antiapoptotic isoform *BCL-XL* could be a potential therapeutic strategy in DN, as it was shown to protect HEK293 cells exposed to a diabetic environment from apoptosis. Finally, the *BCL-XS/BCL-XL* ratio was found to correlate with renal function measures, eGFR and uACR, and may serve as a potential predictive tool for DN severity.

## Author Contributions

**Megan Stevens:** conception and design, acquisition of data, analysis and interpretation of data, drafting the article, final approval. **Monica L. Ayine:** acquisition of data, analysis and interpretation of data, drafting the article, final approval. **Kim Gooding:** conception and design, reviewing the article, final approval. **Angela Shore:** conception and design, reviewing the article, final approval. **Pedro Marqueti:** acquisition of data, reviewing the article, final approval. **Sebastian Oltean:** conception and design, analysis and interpretation of data, drafting the article, final approval. **Megan Stevens** and **Monica L. Ayine** share first authorship.

## Funding

This study was supported by a grant from Diabetes UK (17/0005668). The BEAt‐DKD project has received funding from the Innovative Medicines Initiative 2 Joint Undertaking (JU) under Grant Agreement (No. 115974) (BEAt‐DKD). This Joint Undertaking receives support from the European Union’s Horizon 2020 Research and Innovation Programme and EFPIA with JDRF. The VIBE study was funded by Diabetes UK (Grant Reference: 16/0005489). The study was also supported by the National Institute for Health Research (NIHR) Exeter Clinical Research Facility.

## Disclosure

This paper was first published as a preprint in the BioRxiv server: www.biorxiv.org/content/10.1101/2025.06.06.658276v1.full.pdf+html. The views expressed are those of the authors and not necessarily those of the NIHR or the Department of Health and Social Care.

## Conflicts of Interest

The authors declare no conflicts of interest.

## Supporting Information

Additional supporting information can be found online in the Supporting Information section.

## Supporting information


**Supporting Information 1** Supporting Figures: Contains additional figures cited throughout the manuscript.

**Supporting Information 2** Supporting Table: Contains sequences of the DNA primers used in this study.

## Data Availability

The data that support the findings of this study are available from the corresponding author upon reasonable request.
