# Supplementary material for: Splice Isoforms of the Apoptosis Gene BCL-X Have Opposing Effects in Diabetic Kidney Disease: Potential Treatment Target and Prognostic Value
Source: J Diabetes Res. 2026 Mar 26;2026:2840106. doi: 10.1155/jdr/2840106 (PMC13140356; doi:10.1155/jdr/2840106)
Supplement: Supplementary file 1 — Supporting Information 1 Supporting Figures: Contains additional figures cited throughout the manuscript. [file JDR-2026-2840106-s002.pptx]

## Slide 1
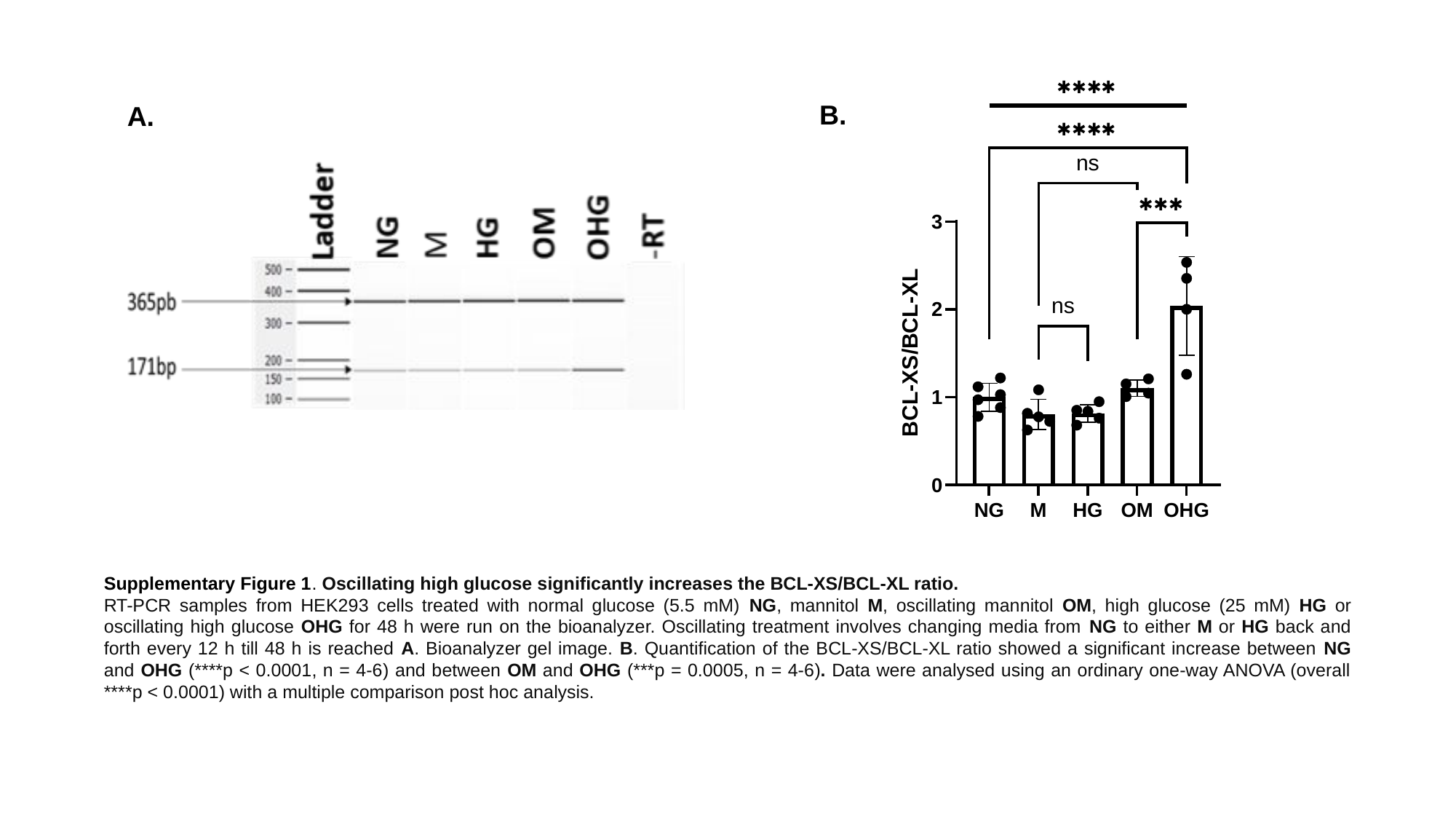

B.
A.
Supplementary Figure 1. Oscillating high glucose significantly increases the BCL-XS/BCL-XL ratio.
RT-PCR samples from HEK293 cells treated with normal glucose (5.5 mM) NG, mannitol M, oscillating mannitol OM, high glucose (25 mM) HG or oscillating high glucose OHG for 48 h were run on the bioanalyzer. Oscillating treatment involves changing media from NG to either M or HG back and forth every 12 h till 48 h is reached A. Bioanalyzer gel image. B. Quantification of the BCL-XS/BCL-XL ratio showed a significant increase between NG and OHG (****p < 0.0001, n = 4-6) and between OM and OHG (***p = 0.0005, n = 4-6). Data were analysed using an ordinary one-way ANOVA (overall ****p < 0.0001) with a multiple comparison post hoc analysis.

## Slide 2
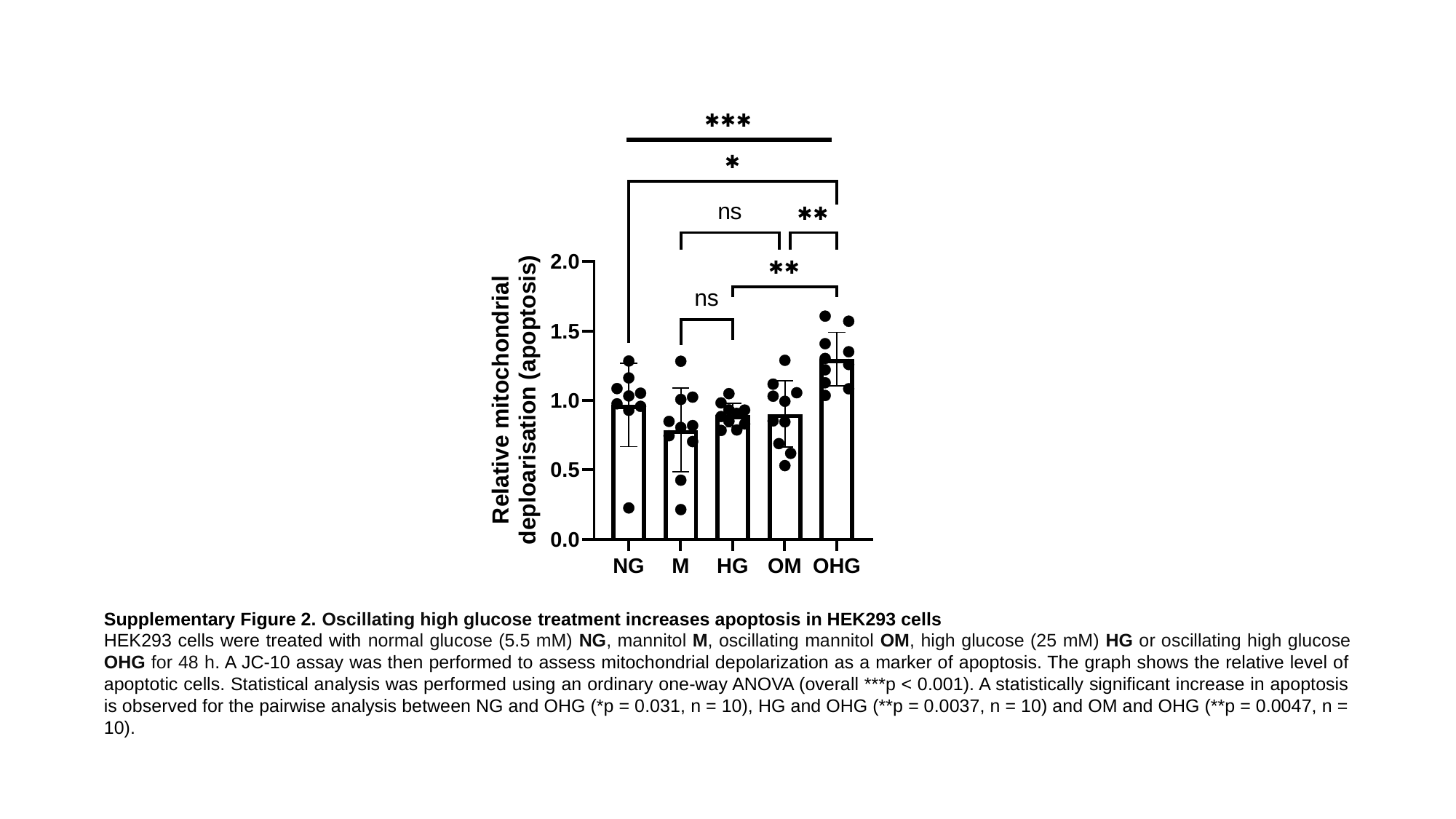

Supplementary Figure 2. Oscillating high glucose treatment increases apoptosis in HEK293 cells
HEK293 cells were treated with normal glucose (5.5 mM) NG, mannitol M, oscillating mannitol OM, high glucose (25 mM) HG or oscillating high glucose OHG for 48 h. A JC-10 assay was then performed to assess mitochondrial depolarization as a marker of apoptosis. The graph shows the relative level of apoptotic cells. Statistical analysis was performed using an ordinary one-way ANOVA (overall ***p < 0.001). A statistically significant increase in apoptosis is observed for the pairwise analysis between NG and OHG (*p = 0.031, n = 10), HG and OHG (**p = 0.0037, n = 10) and OM and OHG (**p = 0.0047, n = 10).

## Slide 3
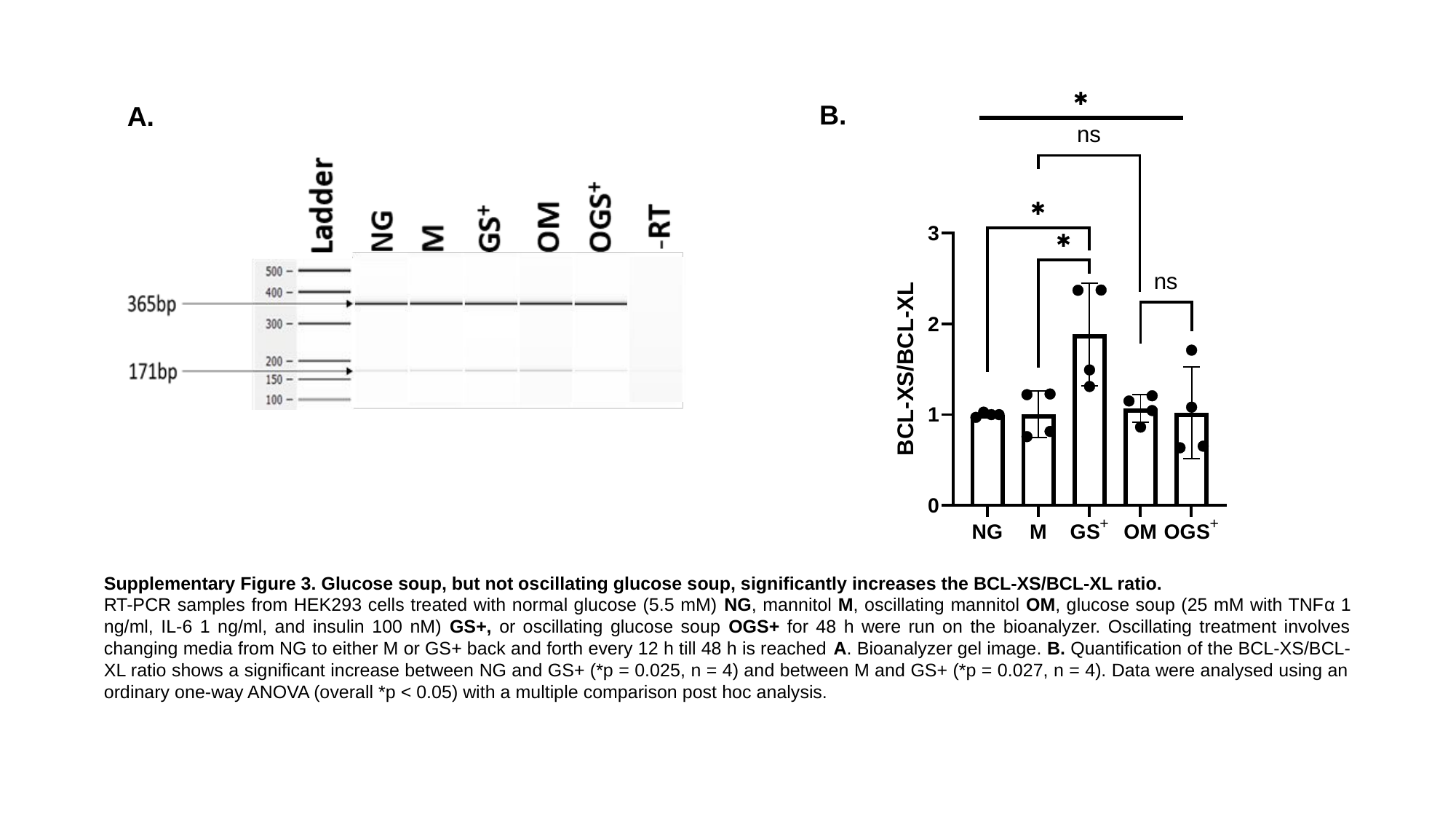

B.
A.
Supplementary Figure 3. Glucose soup, but not oscillating glucose soup, significantly increases the BCL-XS/BCL-XL ratio.
RT-PCR samples from HEK293 cells treated with normal glucose (5.5 mM) NG, mannitol M, oscillating mannitol OM, glucose soup (25 mM with TNFα 1 ng/ml, IL-6 1 ng/ml, and insulin 100 nM) GS+, or oscillating glucose soup OGS+ for 48 h were run on the bioanalyzer. Oscillating treatment involves changing media from NG to either M or GS+ back and forth every 12 h till 48 h is reached A. Bioanalyzer gel image. B. Quantification of the BCL-XS/BCL-XL ratio shows a significant increase between NG and GS+ (*p = 0.025, n = 4) and between M and GS+ (*p = 0.027, n = 4). Data were analysed using an ordinary one-way ANOVA (overall *p < 0.05) with a multiple comparison post hoc analysis.

## Slide 4
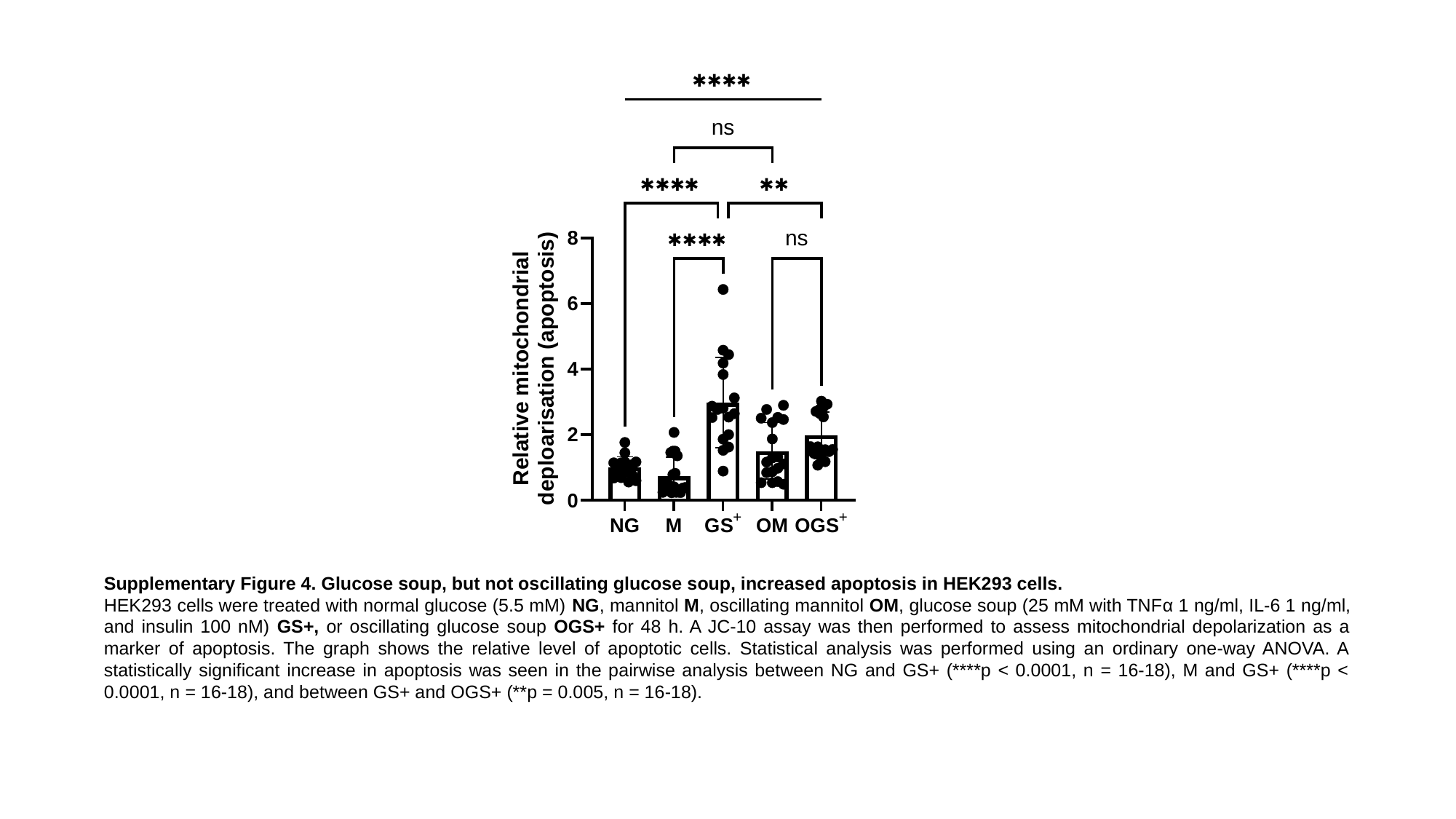

Supplementary Figure 4. Glucose soup, but not oscillating glucose soup, increased apoptosis in HEK293 cells.
HEK293 cells were treated with normal glucose (5.5 mM) NG, mannitol M, oscillating mannitol OM, glucose soup (25 mM with TNFα 1 ng/ml, IL-6 1 ng/ml, and insulin 100 nM) GS+, or oscillating glucose soup OGS+ for 48 h. A JC-10 assay was then performed to assess mitochondrial depolarization as a marker of apoptosis. The graph shows the relative level of apoptotic cells. Statistical analysis was performed using an ordinary one-way ANOVA. A statistically significant increase in apoptosis was seen in the pairwise analysis between NG and GS+ (****p < 0.0001, n = 16-18), M and GS+ (****p < 0.0001, n = 16-18), and between GS+ and OGS+ (**p = 0.005, n = 16-18).

## Slide 5
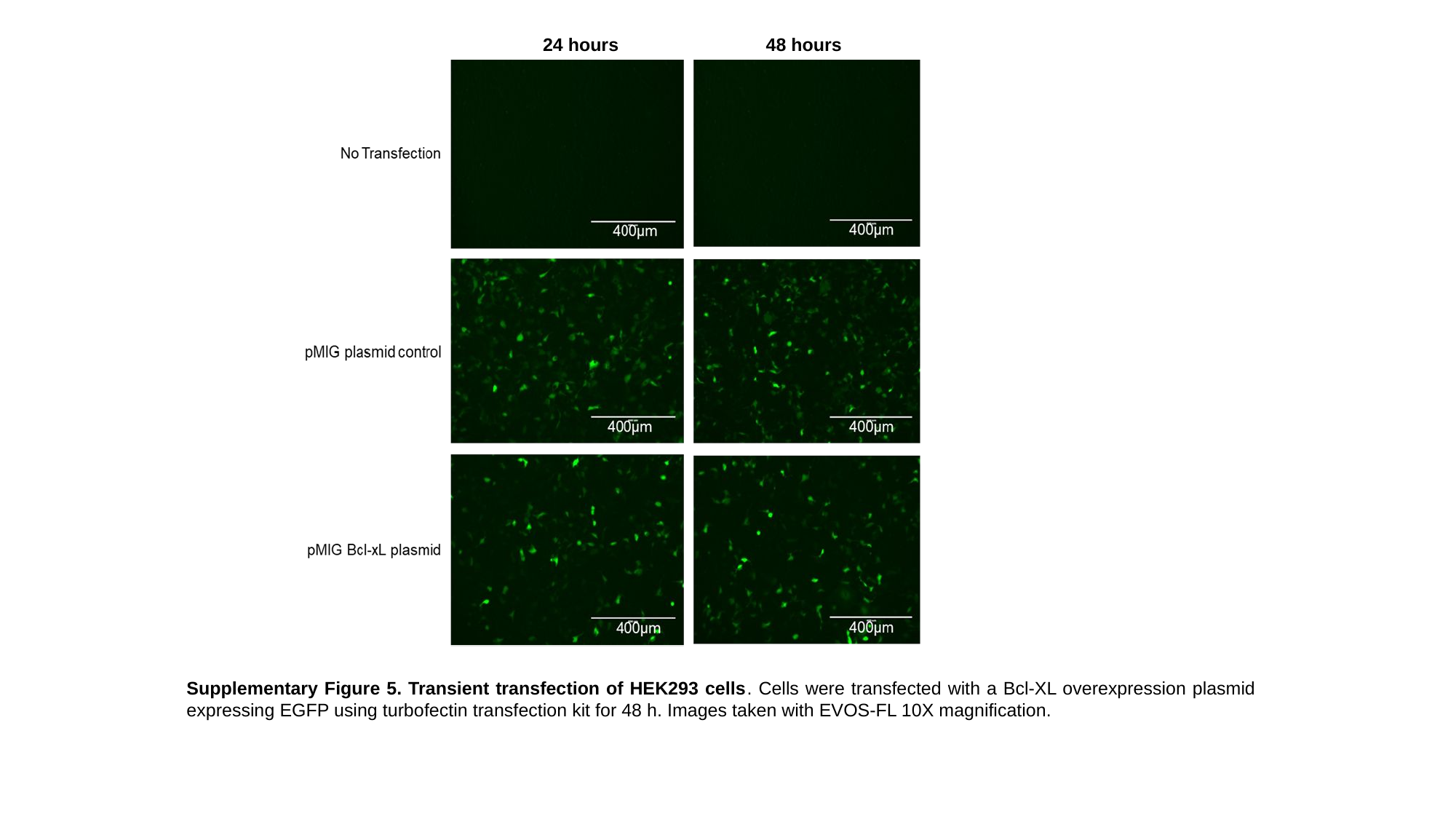

24 hours 48 hours
Supplementary Figure 5. Transient transfection of HEK293 cells. Cells were transfected with a Bcl-XL overexpression plasmid expressing EGFP using turbofectin transfection kit for 48 h. Images taken with EVOS-FL 10X magnification.

## Slide 6
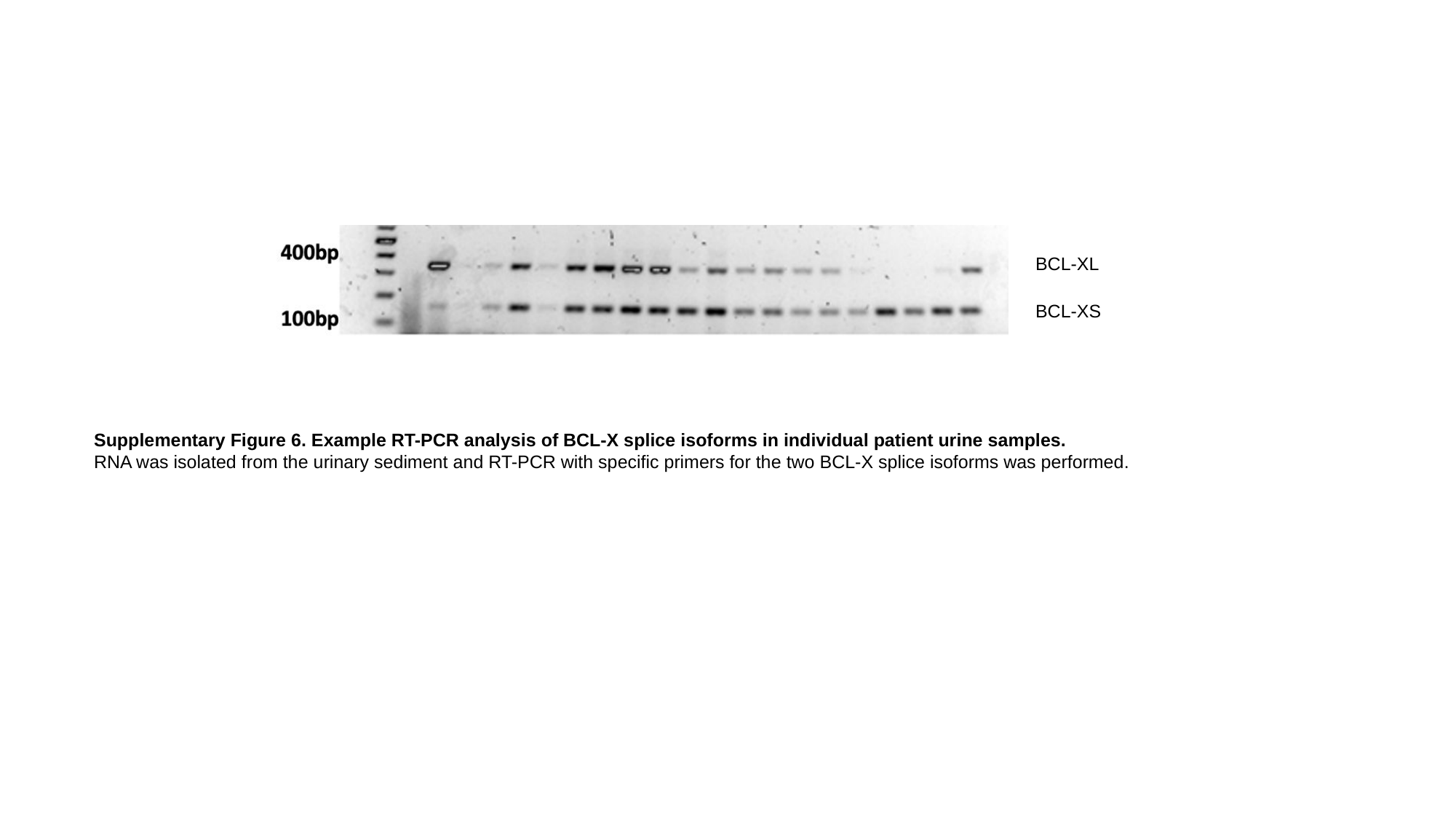

BCL-XL
BCL-XS
Supplementary Figure 6. Example RT-PCR analysis of BCL-X splice isoforms in individual patient urine samples.
RNA was isolated from the urinary sediment and RT-PCR with specific primers for the two BCL-X splice isoforms was performed.

## Slide 7
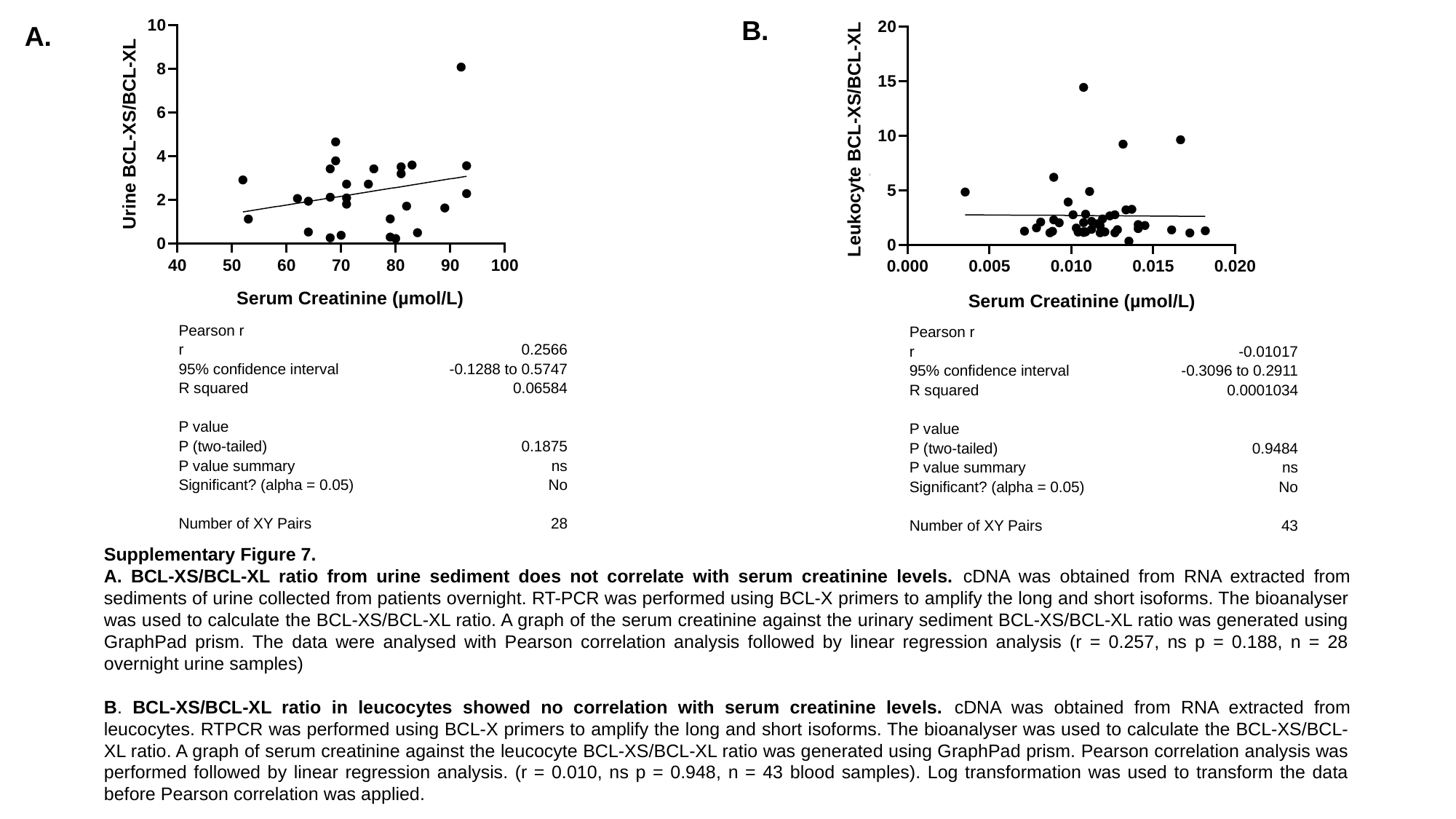

B.
A.
Urine BCL-XS/BCL-XL
Leukocyte BCL-XS/BCL-XL
Serum Creatinine (µmol/L)
Serum Creatinine (µmol/L)
| Pearson r | |
| --- | --- |
| r | 0.2566 |
| 95% confidence interval | -0.1288 to 0.5747 |
| R squared | 0.06584 |
| | |
| P value | |
| P (two-tailed) | 0.1875 |
| P value summary | ns |
| Significant? (alpha = 0.05) | No |
| | |
| Number of XY Pairs | 28 |
| Pearson r | |
| --- | --- |
| r | -0.01017 |
| 95% confidence interval | -0.3096 to 0.2911 |
| R squared | 0.0001034 |
| | |
| P value | |
| P (two-tailed) | 0.9484 |
| P value summary | ns |
| Significant? (alpha = 0.05) | No |
| | |
| Number of XY Pairs | 43 |
Supplementary Figure 7.
A. BCL-XS/BCL-XL ratio from urine sediment does not correlate with serum creatinine levels. cDNA was obtained from RNA extracted from sediments of urine collected from patients overnight. RT-PCR was performed using BCL-X primers to amplify the long and short isoforms. The bioanalyser was used to calculate the BCL-XS/BCL-XL ratio. A graph of the serum creatinine against the urinary sediment BCL-XS/BCL-XL ratio was generated using GraphPad prism. The data were analysed with Pearson correlation analysis followed by linear regression analysis (r = 0.257, ns p = 0.188, n = 28 overnight urine samples)
B. BCL-XS/BCL-XL ratio in leucocytes showed no correlation with serum creatinine levels. cDNA was obtained from RNA extracted from leucocytes. RTPCR was performed using BCL-X primers to amplify the long and short isoforms. The bioanalyser was used to calculate the BCL-XS/BCL-XL ratio. A graph of serum creatinine against the leucocyte BCL-XS/BCL-XL ratio was generated using GraphPad prism. Pearson correlation analysis was performed followed by linear regression analysis. (r = 0.010, ns p = 0.948, n = 43 blood samples). Log transformation was used to transform the data before Pearson correlation was applied.

## Slide 8
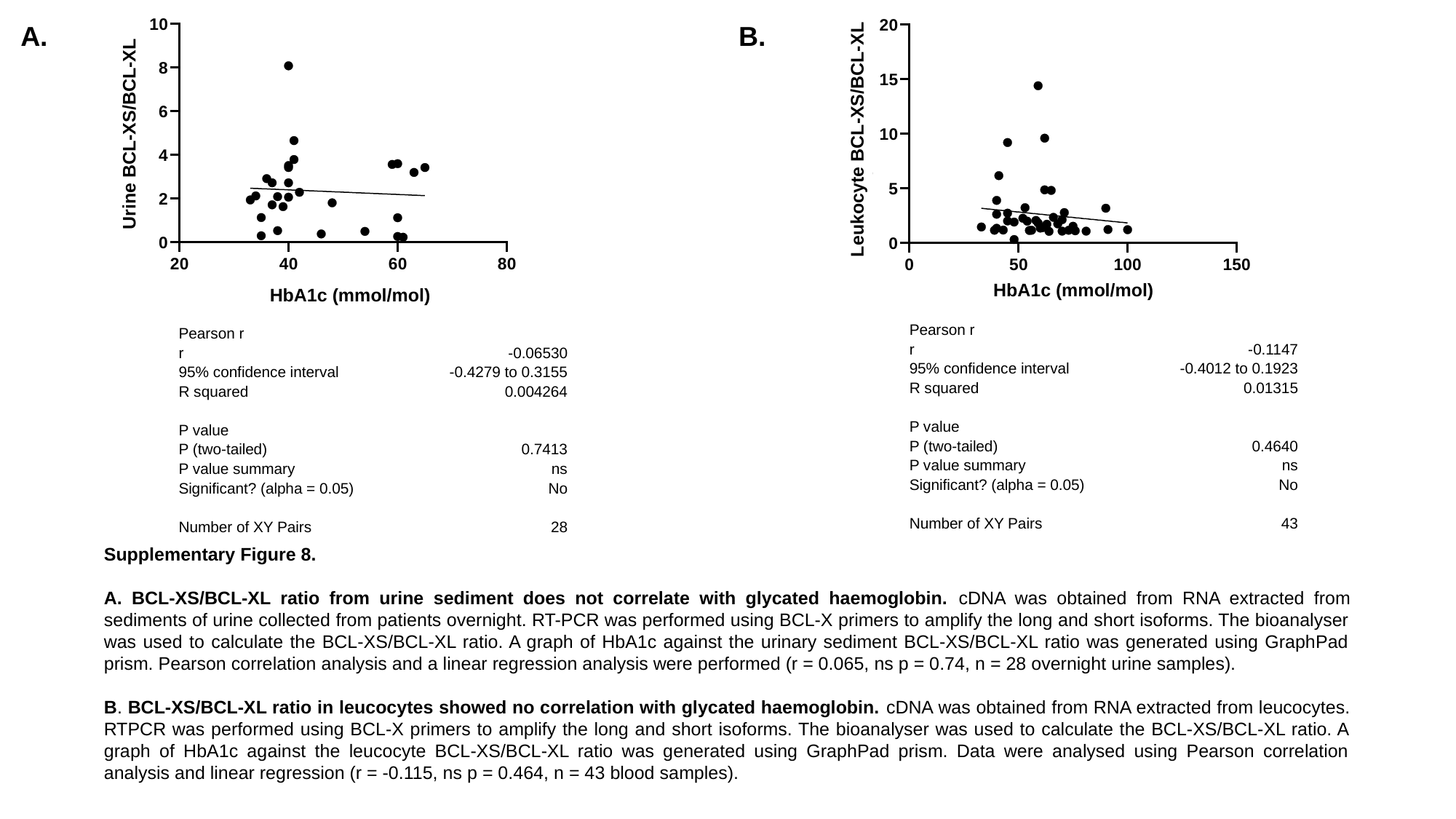

A.
B.
Urine BCL-XS/BCL-XL
Leukocyte BCL-XS/BCL-XL
HbA1c (mmol/mol)
| Pearson r | |
| --- | --- |
| r | -0.1147 |
| 95% confidence interval | -0.4012 to 0.1923 |
| R squared | 0.01315 |
| | |
| P value | |
| P (two-tailed) | 0.4640 |
| P value summary | ns |
| Significant? (alpha = 0.05) | No |
| | |
| Number of XY Pairs | 43 |
| Pearson r | |
| --- | --- |
| r | -0.06530 |
| 95% confidence interval | -0.4279 to 0.3155 |
| R squared | 0.004264 |
| | |
| P value | |
| P (two-tailed) | 0.7413 |
| P value summary | ns |
| Significant? (alpha = 0.05) | No |
| | |
| Number of XY Pairs | 28 |
Supplementary Figure 8.
A. BCL-XS/BCL-XL ratio from urine sediment does not correlate with glycated haemoglobin. cDNA was obtained from RNA extracted from sediments of urine collected from patients overnight. RT-PCR was performed using BCL-X primers to amplify the long and short isoforms. The bioanalyser was used to calculate the BCL-XS/BCL-XL ratio. A graph of HbA1c against the urinary sediment BCL-XS/BCL-XL ratio was generated using GraphPad prism. Pearson correlation analysis and a linear regression analysis were performed (r = 0.065, ns p = 0.74, n = 28 overnight urine samples).
B. BCL-XS/BCL-XL ratio in leucocytes showed no correlation with glycated haemoglobin. cDNA was obtained from RNA extracted from leucocytes. RTPCR was performed using BCL-X primers to amplify the long and short isoforms. The bioanalyser was used to calculate the BCL-XS/BCL-XL ratio. A graph of HbA1c against the leucocyte BCL-XS/BCL-XL ratio was generated using GraphPad prism. Data were analysed using Pearson correlation analysis and linear regression (r = -0.115, ns p = 0.464, n = 43 blood samples).

## Slide 9
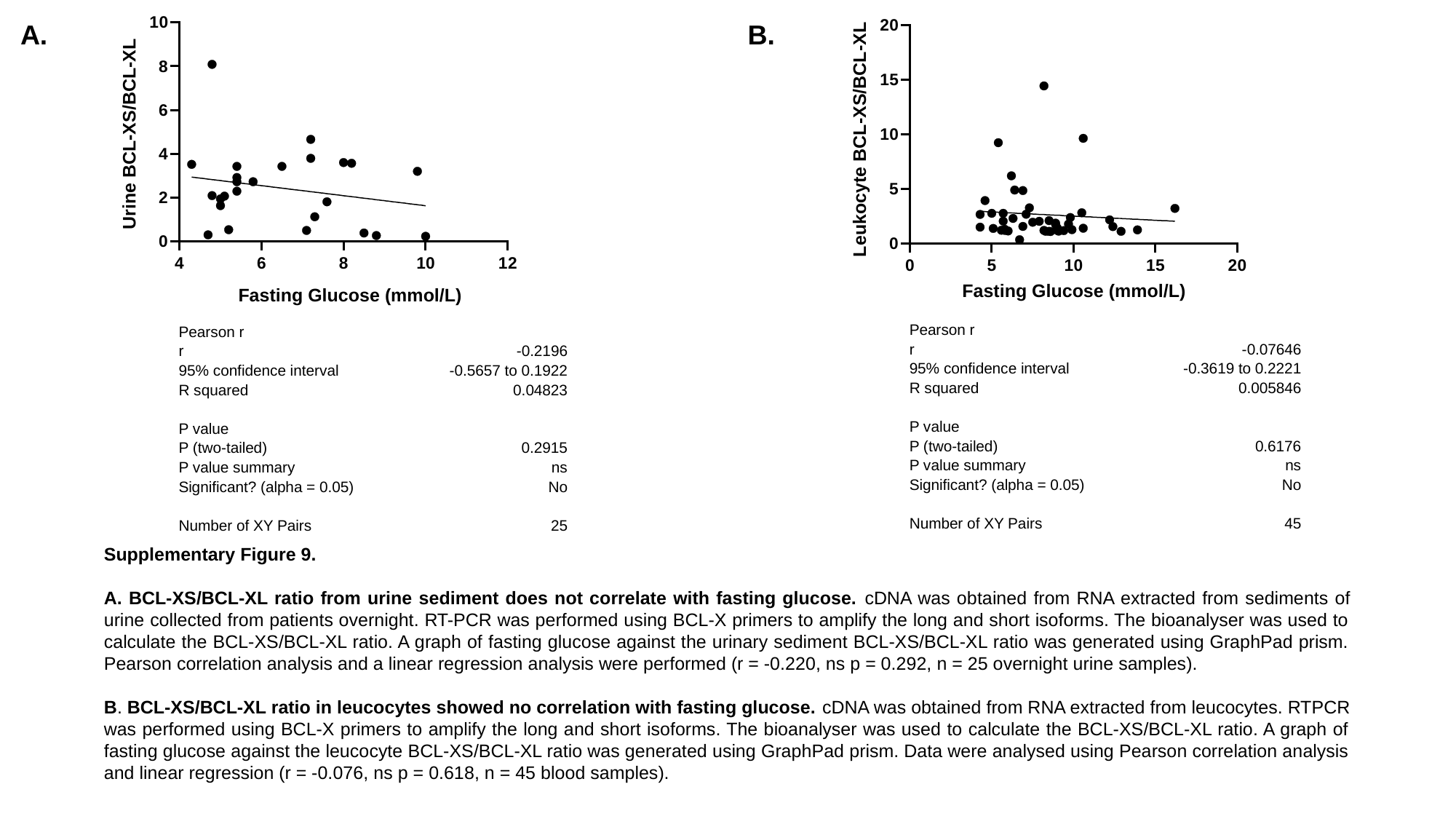

A.
B.
Urine BCL-XS/BCL-XL
Leukocyte BCL-XS/BCL-XL
Fasting Glucose (mmol/L)
| Pearson r | |
| --- | --- |
| r | -0.07646 |
| 95% confidence interval | -0.3619 to 0.2221 |
| R squared | 0.005846 |
| | |
| P value | |
| P (two-tailed) | 0.6176 |
| P value summary | ns |
| Significant? (alpha = 0.05) | No |
| | |
| Number of XY Pairs | 45 |
| Pearson r | |
| --- | --- |
| r | -0.2196 |
| 95% confidence interval | -0.5657 to 0.1922 |
| R squared | 0.04823 |
| | |
| P value | |
| P (two-tailed) | 0.2915 |
| P value summary | ns |
| Significant? (alpha = 0.05) | No |
| | |
| Number of XY Pairs | 25 |
Supplementary Figure 9.
A. BCL-XS/BCL-XL ratio from urine sediment does not correlate with fasting glucose. cDNA was obtained from RNA extracted from sediments of urine collected from patients overnight. RT-PCR was performed using BCL-X primers to amplify the long and short isoforms. The bioanalyser was used to calculate the BCL-XS/BCL-XL ratio. A graph of fasting glucose against the urinary sediment BCL-XS/BCL-XL ratio was generated using GraphPad prism. Pearson correlation analysis and a linear regression analysis were performed (r = -0.220, ns p = 0.292, n = 25 overnight urine samples).
B. BCL-XS/BCL-XL ratio in leucocytes showed no correlation with fasting glucose. cDNA was obtained from RNA extracted from leucocytes. RTPCR was performed using BCL-X primers to amplify the long and short isoforms. The bioanalyser was used to calculate the BCL-XS/BCL-XL ratio. A graph of fasting glucose against the leucocyte BCL-XS/BCL-XL ratio was generated using GraphPad prism. Data were analysed using Pearson correlation analysis and linear regression (r = -0.076, ns p = 0.618, n = 45 blood samples).

## Slide 10
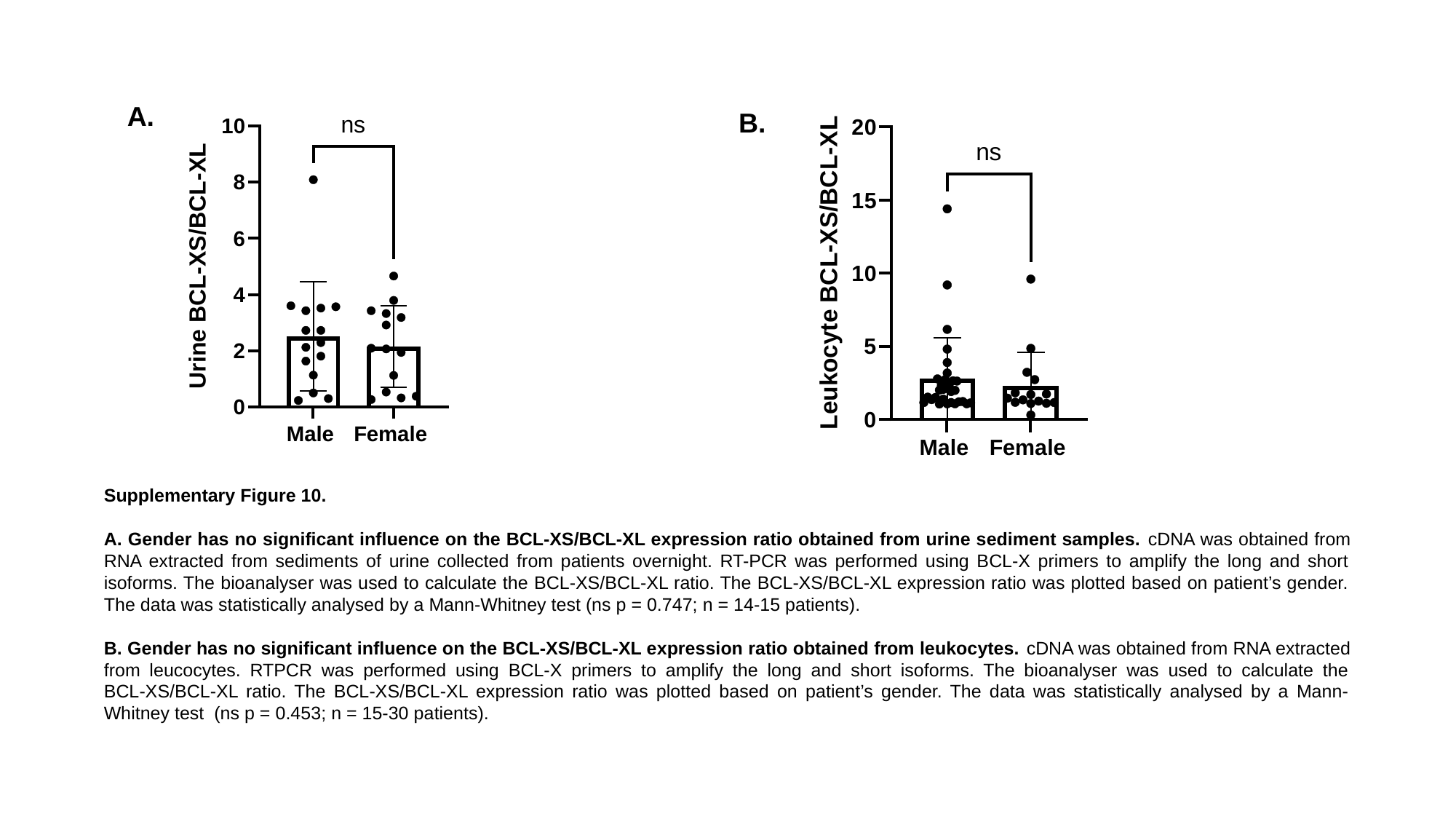

A.
B.
Supplementary Figure 10.
A. Gender has no significant influence on the BCL-XS/BCL-XL expression ratio obtained from urine sediment samples. cDNA was obtained from RNA extracted from sediments of urine collected from patients overnight. RT-PCR was performed using BCL-X primers to amplify the long and short isoforms. The bioanalyser was used to calculate the BCL-XS/BCL-XL ratio. The BCL-XS/BCL-XL expression ratio was plotted based on patient’s gender. The data was statistically analysed by a Mann-Whitney test (ns p = 0.747; n = 14-15 patients).
B. Gender has no significant influence on the BCL-XS/BCL-XL expression ratio obtained from leukocytes. cDNA was obtained from RNA extracted from leucocytes. RTPCR was performed using BCL-X primers to amplify the long and short isoforms. The bioanalyser was used to calculate the BCL-XS/BCL-XL ratio. The BCL-XS/BCL-XL expression ratio was plotted based on patient’s gender. The data was statistically analysed by a Mann-Whitney test (ns p = 0.453; n = 15-30 patients).

## Slide 11
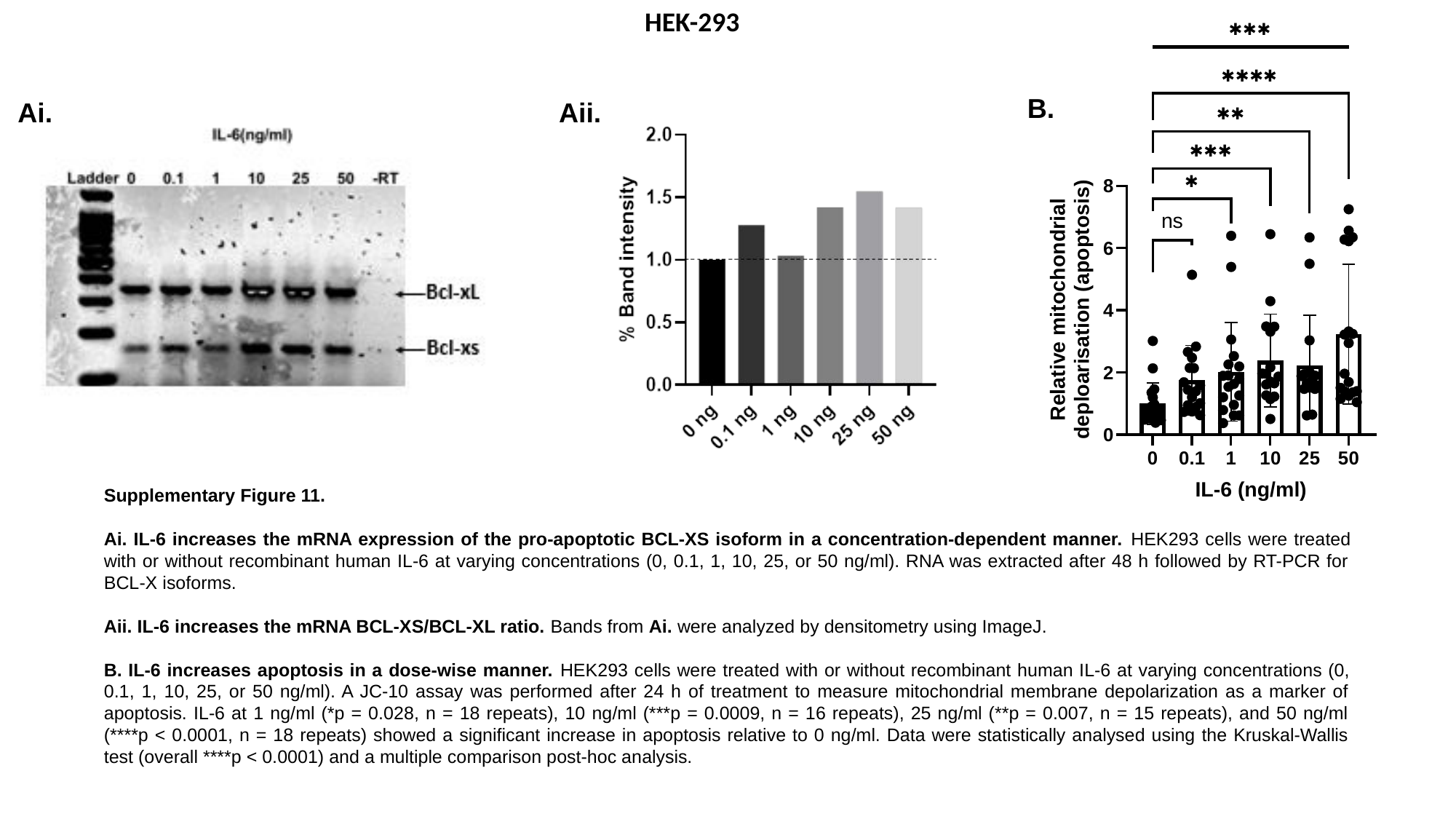

HEK-293
B.
Ai.
Aii.
Supplementary Figure 11.
Ai. IL-6 increases the mRNA expression of the pro-apoptotic BCL-XS isoform in a concentration-dependent manner. HEK293 cells were treated with or without recombinant human IL-6 at varying concentrations (0, 0.1, 1, 10, 25, or 50 ng/ml). RNA was extracted after 48 h followed by RT-PCR for BCL-X isoforms.
Aii. IL-6 increases the mRNA BCL-XS/BCL-XL ratio. Bands from Ai. were analyzed by densitometry using ImageJ.
B. IL-6 increases apoptosis in a dose-wise manner. HEK293 cells were treated with or without recombinant human IL-6 at varying concentrations (0, 0.1, 1, 10, 25, or 50 ng/ml). A JC-10 assay was performed after 24 h of treatment to measure mitochondrial membrane depolarization as a marker of apoptosis. IL-6 at 1 ng/ml (*p = 0.028, n = 18 repeats), 10 ng/ml (***p = 0.0009, n = 16 repeats), 25 ng/ml (**p = 0.007, n = 15 repeats), and 50 ng/ml (****p < 0.0001, n = 18 repeats) showed a significant increase in apoptosis relative to 0 ng/ml. Data were statistically analysed using the Kruskal-Wallis test (overall ****p < 0.0001) and a multiple comparison post-hoc analysis.

## Slide 12
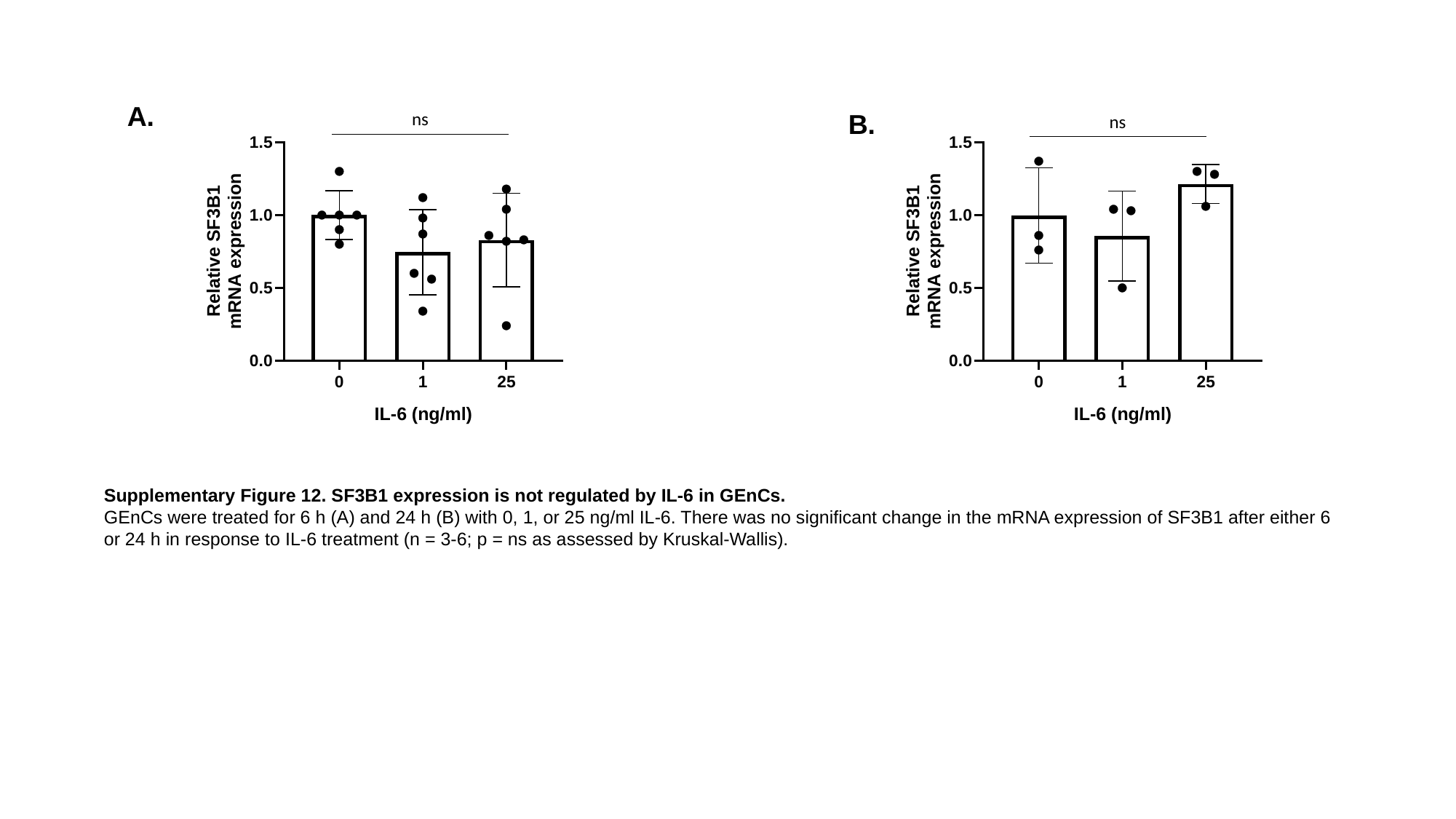

A.
B.
ns
ns
Supplementary Figure 12. SF3B1 expression is not regulated by IL-6 in GEnCs.
GEnCs were treated for 6 h (A) and 24 h (B) with 0, 1, or 25 ng/ml IL-6. There was no significant change in the mRNA expression of SF3B1 after either 6 or 24 h in response to IL-6 treatment (n = 3-6; p = ns as assessed by Kruskal-Wallis).
